# Molecular mechanism of *SmMYB53* activates the expression of *SmCYP71D375*, thereby modulating tanshinone accumulation in *Salvia miltiorrhiza*

**DOI:** 10.1093/hr/uhaf058

**Published:** 2025-02-27

**Authors:** Xinyu Wang, Yifei Shi, Qichao Wang, Xinjia Xie, Siqi Gui, Jiening Wu, Limei Zhao, Xiaowei Zou, Guoyin Kai, Wei Zhou

**Affiliations:** Laboratory for Core Technology of TCM Quality Improvement and Transformation, School of Pharmaceutical Sciences, School of Pharmacy and Academy of Chinese Medical Science, Zhejiang Chinese Medical University, Hangzhou 310053, China; Laboratory for Core Technology of TCM Quality Improvement and Transformation, School of Pharmaceutical Sciences, School of Pharmacy and Academy of Chinese Medical Science, Zhejiang Chinese Medical University, Hangzhou 310053, China; Laboratory for Core Technology of TCM Quality Improvement and Transformation, School of Pharmaceutical Sciences, School of Pharmacy and Academy of Chinese Medical Science, Zhejiang Chinese Medical University, Hangzhou 310053, China; Laboratory for Core Technology of TCM Quality Improvement and Transformation, School of Pharmaceutical Sciences, School of Pharmacy and Academy of Chinese Medical Science, Zhejiang Chinese Medical University, Hangzhou 310053, China; Laboratory for Core Technology of TCM Quality Improvement and Transformation, School of Pharmaceutical Sciences, School of Pharmacy and Academy of Chinese Medical Science, Zhejiang Chinese Medical University, Hangzhou 310053, China; Laboratory for Core Technology of TCM Quality Improvement and Transformation, School of Pharmaceutical Sciences, School of Pharmacy and Academy of Chinese Medical Science, Zhejiang Chinese Medical University, Hangzhou 310053, China; Laboratory for Core Technology of TCM Quality Improvement and Transformation, School of Pharmaceutical Sciences, School of Pharmacy and Academy of Chinese Medical Science, Zhejiang Chinese Medical University, Hangzhou 310053, China; Laboratory for Core Technology of TCM Quality Improvement and Transformation, School of Pharmaceutical Sciences, School of Pharmacy and Academy of Chinese Medical Science, Zhejiang Chinese Medical University, Hangzhou 310053, China; Laboratory for Core Technology of TCM Quality Improvement and Transformation, School of Pharmaceutical Sciences, School of Pharmacy and Academy of Chinese Medical Science, Zhejiang Chinese Medical University, Hangzhou 310053, China; Laboratory for Core Technology of TCM Quality Improvement and Transformation, School of Pharmaceutical Sciences, School of Pharmacy and Academy of Chinese Medical Science, Zhejiang Chinese Medical University, Hangzhou 310053, China

## Abstract

Tanshinones are bioactive diterpenoid chemicals of the herb *Salvia miltiorrhiza* with a characteristic furan D-ring. As a newly identified downstream enzyme, SmCYP71D375, catalyzes hydroxylation by 14,16-ether (hetero)cyclization to form the furan D-ring from the precursor of the phenolic abietane-type diterpenoids that exist widely in Lamiaceae plants. However, its transcriptional regulatory network, with *SmCYP71D375* as the direct target gene, remains unclear. In the present study, the promoter of *SmCYP71D375* was employed as the bait to mine the upstream regulatory protein using the cDNA yeast library of *S. miltiorrhiza*. An R2R3-MYB transcription factor gene, *SmMYB53*, was identified. Overexpressing *SmMYB53* in transgenic hairy roots upregulated *SmCYP71D375* expression, thereby accelerating tanshinone accumulation, whereas tanshinone accumulation was inhibited in *SmMYB53-*RNAi transgenic hairy root lines. To dissect the regulatory network of *SmMYB53*, *SmbZIP51* was captured using *SmMYB53* as the bait to prey for its potential interacting proteins in the cDNA yeast library. Yeast two-hybrid, glutathione *S*-transferase pull-down, and bimolecular fluorescence complementation assays were independently used to verify the interaction between the SmMYB53 and SmbZIP51 proteins . We further verified that the upregulation of *SmCYP71D375* activated by SmMYB53 would be inhibited by the interaction of SmMYB53 and SmbZIP51. The present findings uncover the molecular regulatory network underlying *SmCYP71D375* as the direct target regulating tanshinone biosynthesis and offer a basis for the genetic improvement of medicinal substance biosynthesis in *S. miltiorrhiza*.

## Introduction

*Salvia miltiorrhiza* is a Chinese medicinal herb whose dried roots and rhizome can be used as raw herbal material. Tanshinones, which are among its main medicinal components, mainly include tanshinone I (TI), tanshinone IIA (TIIA), cryptotanshinone (CT), and dihydrotanshinone (DT) [1]. They have antitumor, anti-inflammatory, and other pharmacological effects [2]. Based on their pharmacological activity, tanshinones are used to treat diverse cardiovascular diseases in the clinic [3]. Nevertheless, obtaining large amounts of tanshinones is difficult due to the plant’s long growth cycle and low tanshinone content in roots. Therefore, how to elevate the tanshinone content in *S. miltiorrhiza* has attracted wide attention. Nowadays, with the continuous progress of genetic manipulation techniques, the biosynthesis pathway of tanshinones and its regulatory network has been gradually elucidated, and it provides a theoretical basis for improving tanshinone accumulation in *S. miltiorrhiza* through genetic manipulation [4].

As diterpenoid compounds, tanshinones are mainly produced through two parallel pathways, the mevalonate (MVA) pathway and the methylerythritol phosphate (MEP) pathway, by which the same intermediates—isopentenyl pyrophosphate (IPP) and dimethylallyl pyrophosphate (DMAPP)—are converted (Supplementary Data Fig. S1). In the MVA pathway, two molecules of acetyl-CoA are catalyzed to synthesize DMAPP and IPP step by step by six catalyzing enzymes [4–7]. In the MEP pathway, pyruvate and glyceraldehydyde-3-phosphate (GA-3P), as two initial reaction substrates, are metabolized to DMAPP and IPP via seven catalytic reaction steps [8, 9]. IPP and DMAPP are subsequently catalyzed by geranyl diphosphate synthase (GPPS), geranylgeranyl diphosphate synthase (GGPPS) [4], copalyl diphosphate synthase (CPS), kaurene synthase-like (KSL) [10], cytochrome P450 (CYP76AH1, CYP76AK1, CYP76AH3, CYP71D375) [11–13], and other unknown catalytic enzymes to synthesize tanshinones. Among them, SmCYP71D375 is a newly identified most downstream catalytic enzyme with the function of catalyzing hydroxylation by 14,16-ether (hetero)cyclization to generate the furan D-ring of tanshinones from the precursor of the phenolic abietane-type diterpenoids that commonly exist in Lamiaceae plants [13].

The regulatory mechanism mediated by certain transcription factors (TFs) with the above genes HMGS, HMGR, DXS1, DXR, GGPPS, CPS1, KSL1 and CYP76AH1 as the direct targets has been previously reported. In *S. miltiorrhiza*, *SmERF1L1* promotes tanshinone biosynthesis by activating *SmDXR* expression [14]. *SmERF106*, as a methyl jasmonate (MeJA)-responsive TF, can activate the transcription of *SmKSL*, thereby accelerating tanshinone biosynthesis in transgenic hairy roots [15]. However, the transcriptional regulatory network with *SmCYP71D375* as the direct target, a downstream gene in the tanshinone biosynthetic pathway, remains unclear. Compared with the precursor of phenolic abietane-type diterpenoids, the furan D-ring structure of tanshinones (e.g. cryptotanshinone and 15,16-dihydrotanshinone) exhibits more powerful pharmacological activities [3, 13], thus highlighting the importance of mining the candidate TFs and dissecting the molecular regulatory network with *SmCYP71D375* as the direct target.

As one of the largest families of TFs in plants, MYB TFs are divided into four classes based on the repeat sequences in the N-terminal MYB domains, namely 1R-, R2R3-, R1R2R3-, and 4R-MYB [16], among which R2R3-MYB is the most widely studied group and is thought to participate in plant secondary metabolism, disease resistance, growth, and development [17]. As an R2R3-MYB member, *SmMYB9b* promotes tanshinone accumulation by stimulating the MEP pathway [18]. *SmMYB36* activates target gene expression by binding to the MBS elements within the *DXS2*, *GGPPS1*, and *CPS1* promoters, thus promoting the synthesis of tanshinones [19]. *OsMYB1* is thought to be related to phosphorus hunger signal transduction and gibberellin biosynthesis to affect root growth [20]. *AtMYB1*, *AtMYB25*, and *AtMYB109* were classified into the S23 subgroup of the R2R3-MYB family, which were validated to regulate salt tolerance, osmotic stress and abscisic acid signaling [21]. *SlMYB1*, in the S23 subgroup of the R2R3-MYB family, not only improves the disease resistance of tomatoes, but also promotes lycopene and flavonoid biosynthesis [22]. By analyzing the *cis*-elements in the *SmCYP71D375* promoter, two MYB protein-characteristic *cis*-elements, MBS1 and MBS2, have been discovered (Supplementary Data Fig. S2). However, whether certain R2R3-MYB members can regulate tanshinone accumulation by directly activating *SmCYP71D375* expression in *S. miltiorrhiza* is still obscure.

In the present study, the *SmCYP71D375* promoter was employed as a bait to prey in the yeast one-hybrid (Y1H) cDNA library of *S. miltiorrhiza*, and SmMYB53 was finally captured. *SmMYB53* is a new member of the S23 subfamily in the R2R3-MYB family. We demonstrated that *SmMYB53* activated *SmCYP71D375* expression and improved tanshinone accumulation. Moreover, SmbZIP51 was validated to interact with SmMYB53 to form a protein module to decrease the expression of the target gene *SmCYP71D375* compared with the action of SmMYB53 alone. In summary, this study offers a new insight into the molecular mechanism of anchoring *SmCYP71D375* as the direct target of *SmMYB53*, as an R2R3-MYB TF, to modulate tanshinone accumulation in *S. miltiorrhiza*.

## Results

### Capture of *SmMYB53* by screening yeast one-hybrid library

Due to the vital role of *SmCYP71D375* involved in tanshinone biosynthesis and the multiple functions of R2R3-MYB TFs in association with plant secondary metabolism, we employed the two MYB binding sites (MBS1:CAGTTG, MBS2:CCGTTG) in the promoter of *SmCYP71D375* to insert the pHIS vector as the bait to conduct yeast one-hybrid (Y1H) library screening. With the MBS1 *cis*-element as the bait, 20 candidate proteins were captured from the Y1H library (Supplementary Data Table S1), whereas we only preyed 13 candidate proteins using the MBS2 *cis*-element as the bait (Supplementary Data Table S2). Merging the above two results, we finally anchored an R2R3 transcription factor, namely *SmMYB53* (NCBI accession number KF059407.1), based on a previous report [17]. *SmMYB53*, which is one of the above 33 preys and was validated previously to participate in terpenoid accumulation in *Arabidopsis thaliana* [23], was chosen for further study.

### Characterization of *SmMYB53*

**Figure 1:**
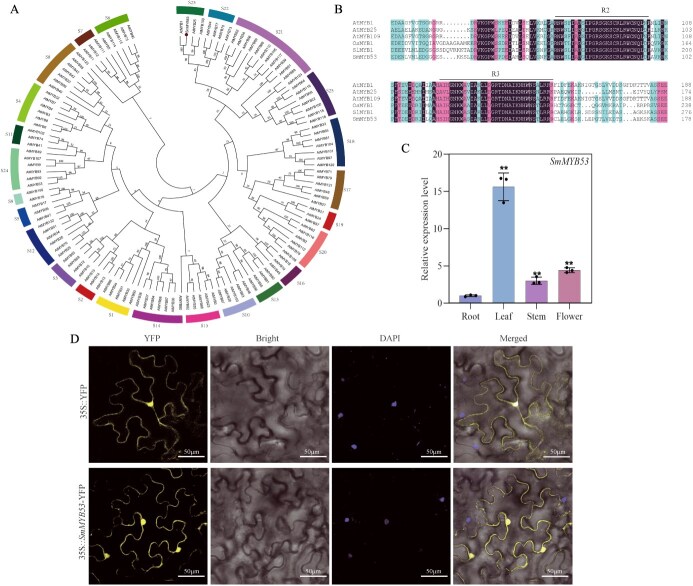
Phylogenetic tree construction, conserved domain, and expression profiles of *SmMYB53*. (A) Phylogenetic tree of *SmMYB53* and 125 R2R3-MYB TFs of *A. thaliana*. (B) Conserved domain of *SmMYB53*. (C) Relative expression level of *SmMYB53* in four tissues of *S. miltiorrhiza*. All detections were repeated three times. Asterisks denote significant difference by the *t*-test (^**^P < 0.01). (D) Subcellular localization of SmMYB53. DAPI was the positive control.

We assembled the open reading fragment (ORF) of *SmMYB53* based on an established transcriptome database. Then, special *SmMYB53* gene primers were introduced to amplify the ORF sequence of *SmMYB53*, which was subjected to sequencing for final validation. The cDNA sequence of *SmMYB53* contained an ORF 1119 bp in length, encoding a total of 372 amino acids with the predicted molecular weight of 39.67 kDa. We constructed a phylogenetic tree of *SmMYB53* protein with 125 MYB members in *A. thaliana*. This result shows that *SmMYB53* belongs to the S23 subgroup together with *AtMYB1*, *AtMYB25*, and *AtMYB109* in *A. thaliana* (Fig. 1A). In order to clarify the structural characteristics of *SmMYB53*, we employed multiple protein sequence alignment of *SmMYB53* and four typical R2R3-MYB TFs including *SlMYB1* (NCBI accession number Solyc09g011780), *AtMYB1* (AT3G09230), *AtMYB25* (AT2G39880), *AtMYB109* (AT3G55730), and *OsMYB1* (LOC4324801) [22, 24]. The result showed that the N-terminal of *SmMYB53* has the highly conserved R2 and R3 domains (Fig. 1B), implying that it belongs to the R2R3 subgroup of MYB TF families.

### Expression profile o*f SmMYB53*

We employed qRT–PCR to examine the *SmMYB53* expression profile in various tissues, namely, leaf, stem, flower, and root. As shown in Fig. 1C, *SmMYB53* was expressed vigorously in the four tissues, and it exhibited the lowest expression level in root and peaked in leaf. Transient expression of SmMYB53 fused with YFP in epidermal cells from *Nicotiana benthamiana* leaves was used to explore the subcellular localization of SmMYB53. We observed distinct fluorescence in the whole cell with the construct of 35S::*SmMYB53*-YFP, while the 35S::YFP control also exhibited robust fluorescence signal in the whole cell, implying that SmMYB53 localizes in the whole cell (Fig. 1D).

### *SmMYB53* promotes tanshinone biosynthesis by upregulating *SmCYP71D375* expression in *S. miltiorrhiza* transgenic hairy roots

**Figure 2:**
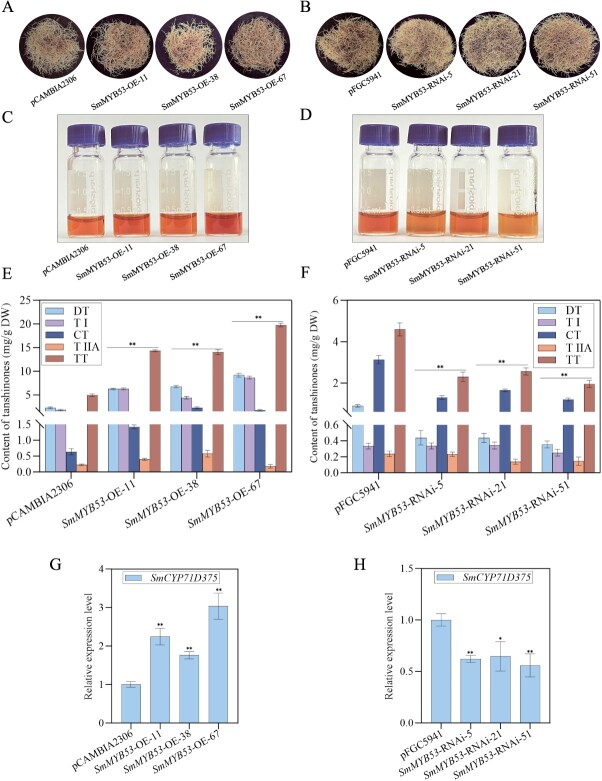
Tanshinone yield and expression profile of *SmCYP71D375* in *SmMYB53* transgenic hairy roots. (A, B) Phenotype of *SmMYB53*-OE lines and *SmMYB53*-RNAi lines. (C, D) Tanshinone extraction. (E, F) Tanshinone content detected by HPLC. (G, H) Expression profile of *SmCYP71D375* in *SmMYB53* transgenic hairy roots. Asterisks denote significant difference at two levels by the *t*-test (^*^P < 0.05, ^**^P < 0.01).

To investigate *SmMYB53* in modulating tanshinone accumulation, we generated *SmMYB53*-flag overexpression (OE) and RNA interference (RNAi) transgenic hairy roots under the control of the CaMV 35S promoter. The *SmMYB53*-OE and *SmMYB53*-RNAi lines were firstly identified by genomic PCR amplification (Supplementary Data Fig. S3A and B). Then, three *SmMYB53*-OE hairy root lines (*SmMYB53*-OE-11, -38, and -67) with the highest expression and three *SmMYB53*-RNAi lines (*SmMYB53*-RNAi-5, -21, and -51) expressed at the lowest level compared with the control were chosen for further analysis (Supplementary Data Fig. S3C and D). A high-performance liquid chromatography (HPLC) assay was used to quantitatively measure tanshinone yield in the independent OE and RNAi lines (Fig. 2A and B). The yields of four tanshinones, including TI, TIIA, CT, and DT, and total tanshinones (TT), were all significantly elevated (Fig. 2C and D). As shown in Fig. 2E, the TT yields in the three *SmMYB53*-OE lines were 2.8-, 2.75-, and 3.91-fold higher than that of the control, respectively. Among them, *SmMYB53*-OE-67 accumulated the highest yield of TT, up to 20 mg/g DW. By contrast, the TT concentrations in three *SmMYB53*-RNAi lines were only 0.5-, 0.56-, and 0.42-fold that of the control, with the lowest TT yield decreased to 1.33 mg/g DW in the *SmMYB53*-RNAi-51 line (Fig. 2F). Moreover, we confirmed that overexpressing *SmMYB53* upregulated the expression of *SmCYP71D37*5, *SmKSL1*, *SmGGPPS1*, and *SmCYP76AH1* in three *SmMYB53*-OE lines (Fig. 2G and Supplementary Data Fig. S4A), but drastically downregulated it in three *SmMYB53*-RNAi lines in comparison with the control (Fig. 2H and Supplementary Data Fig. S4B). These findings demonstrate that *SmMYB53* upregulates four genes (*SmCYP71D37*5, *SmKSL1*, *SmGGPPS1*, and *SmCYP76AH1*) in the tanshinone biosynthesis pathway, thereby promoting tanshinone biosynthesis in *S. miltiorrhiza* transgenic hairy roots.

### SmMYB53 binds to the *SmCYP71D375* promoter

**Figure 3:**
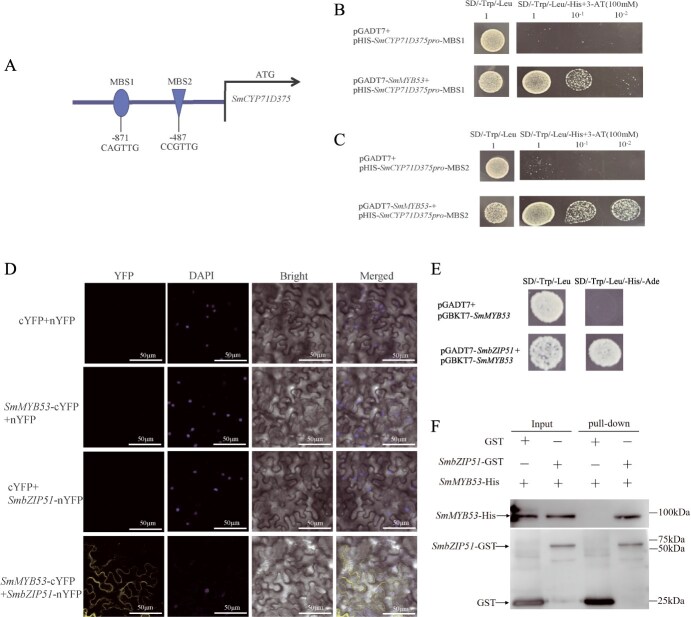
Validation of SmMYB53 binding to the *SmCYP71D375* promoter and interaction of SmbZIP51 with SmMYB53. (A) Schematic diagram of MYB *cis*-elements present in *SmCYP71D375* promoter. (B, C) Y1H assays. (D) BiFC experiment. Scale bars represent 50 μm. (E) Y2H experiment. (F) Pull-down experiment.

Y1H was used to further validate SmMYB53 binding to the *SmCYP71D375* promoter. The Y1H assay showed that SmMYB53 could bind to the MBS1 (CAGTTG) and MBS2 (CCGTTG) *cis*-elements in the *SmCYP71D375* promoter (Fig. 3A–C). Collectively, these results substantiate that SmMYB53 binds to the MBS1 and MBS2 *cis*-elements within the *SmCYP71D375* promoter.

### Validation of interaction between SmMYB53 and SmbZIP51

To explore the potential regulatory network modulated by SmMYB53, we employed the yeast two-hybrid (Y2H) assay to mine the candidate interacting proteins related to tanshinone biosynthesis. With pGBKT7-*SmMYB53* as the bait, we obtained 36 prey sequences from a cDNA yeast library (Supplementary Data Table S3). Among them, a bZIP transcription factor designated SmbZIP51 showed higher capture frequency than other protein preys, and was reported to participate in ABA signaling, drought stress, and biotic and abiotic stress of *A. thaliana* [25]. Therefore, we performed a bimolecular fluorescent complementation (BiFC) assay, Y2H, and pull-down experiments to further verify the interaction of SmMYB53 and SmbZIP51. The BiFC assay verified the interactions of SmMYB53 and SmbZIP51 *in vivo*. As shown in Fig. 3D, a robust fluorescence signal was visible when SmMYB53-cYFP and SmbZIP51-nYFP were coexpressed in *N. benthamiana* leaves, but there was no signal with cYFP or nYFP alone as negative controls. Then, a Y2H assay showed that yeast cells harboring recombinants of pGADT7-*SmbZIP51* and pGBKT7-*SmMYB53* grew normally on SD medium without Trp, Leu, His, and Ade (QDO), thereby validating the interaction of SmMYB53 and SmbZIP51 (Fig. 3E). Moreover, we also confirmed that glutathione *S*-transferase (GST)-tagged SmbZIP51 could bind specifically to His-tagged SmMYB53 by the pull-down assay (Fig. 3F).

### SmbZIP51 interacts with SmMYB53 to inhibit its activation of the expression of *SmCYP71D375*

**Figure 4:**
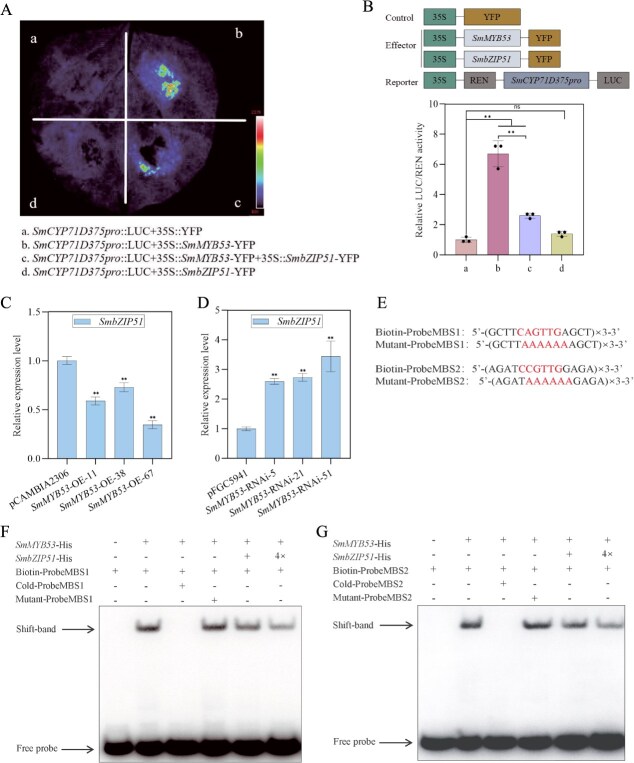
SmbZIP51 interacts with SmMYB53 to inhibit its activation of the expression of *SmCYP71D375*. (A, B) Coexpressing *SmbZIP51* and *SmMYB53* in *N. benthamiana* leaves to inhibit its activation of the expression of *SmCYP71D375* than the action of *SmMYB53* alone, as validated by dual-LUC experiments. Significant differences were determined by the *t*-test (^**^P < 0.01). All the experiments were replicated three times. (C, D) Expression profile of *SmbZIP51* in *SmMYB53* transgenic hairy roots. Asterisks denote significant differences by the *t*-test (^**^P < 0.01). (E) Probes for EMSA assays. (F, G) EMSA assays. The cold probes were identical to the biotin-labeled probes but without biotin labels. The mutant probes were without biotin labels.

We employed a dual-LUC experiment to evaluate whether SmMYB53 modulates the transcription of genes participating in the medicinal substance biosynthesis pathway by using transient gene expression in *N. benthamiana* leaves. As shown in Fig. 4A and B, SmMYB53 only activated the *SmCYP71D375* promoter with a 6.7-fold increase compared with the control, and no significant variation was observed for other reporter constructs (Supplementary Data Fig. S5A, B). To confirm whether SmMYB53 together with SmbZIP51 additively activate *SmCYP71D375* expression to a higher degree than either of them alone, we employed a dual-LUC assay to examine the LUC/REN reporter activity driven by the *SmCYP71D375* promoter, which was activated by the effectors of SmMYB53 and SmbZIP51 alone or simultaneously. Notably, no activation was examined with *SmCYP71D375* as the reporter and SmbZIP51 as the effector, whereas *SmCYP71D375* promoter activation drastically decreased when SmMYB53 was coexpressed together with SmbZIP51 compared with SmMYB53 alone (Fig. 4A and B). Moreover, we found that overexpressing *SmMYB53* downregulated the expression of *SmbZIP51* in three *SmMYB53*-OE lines (Fig. 4C), but drastically upregulated it in three *SmMYB53*-RNAi lines in comparison with the control (Fig. 4D).

We employed electrophoretic mobility shift assay (EMSA) experiments with a complement of both SmbZIP51 and SmMYB53 proteins and SmMYB53 alone to validate the target gene *SmCYP71D375*. As shown in Fig. 4E–G, SmbZIP51 interacts with SmMYB53 to inhibit the binding ability of SmMYB53 to the biotin-labeled MBS1 and MBS2 probes, as the brightness of the shift band in the SmbZIP51 + SmMYB53 lane is significantly weaker than that in the SmMYB53 alone lane.

## Discussion

### *SmMYB53* is the earliest identified regulator with *SmCYP71D375* as the target gene in promoting tanshinone biosynthesis in *S. miltiorrhiza*

With the promoters of target genes as bait, many important TFs were captured through yeast library screening [22, 24]. To mine TFs related to regulation of the sweetness of melon, the promoter of *CmSPS1* (sucrose phosphate synthase) participating in the sucrose synthesis pathway was employed as bait to conduct yeast library screening, and *CmMYB44* was preyed. Further experiments verified that *CmMYB44* regulated the sweetness of melon by activating *CmSPS1* expression [26]. In grape, using the W-box (TTGAC) element as bait, *VvWRKY70* was captured from the yeast library, and the dual-LUC experiment further demonstrated that *VvWRKY70* decreased noroprene and flavonol accumulation by binding to the promoter of the target gene to inhibit noroprene and flavonol biosynthesis [27]. Thus, the above research strategy enabled us to capture the candidate *SmMYB53* transcription factor successfully with the MBS1 and MBS2 *cis*-elements in the *SmCYP71D375* promoter as the bait to prey the yeast library of *S. miltiorrhiza*. To our knowledge, *SmMYB53* is the first identified regulator with *SmCYP71D375* as the target gene in promoting tanshinone biosynthesis in *S. miltiorrhiza* hairy roots.

Moreover, two other TFs involved in nitrogen signaling, annotated as NLP6-like (NCBI accession number XM_057945137.1) and nitrate regulatory gene 2 protein-like (NCBI accession number XM_057952347.1), were also preyed based on yeast library screening with MBS1 or MBS2 as the bait (Supplementary Data Tables S1 and S2). Whether the above two TFs can mediate in nitrogen signaling and bind to the promoter of *SmCYP71D375* to participate in tanshinone biosynthesis remains obscure. Nitrogen is an irreplaceable nutrient element in promoting the growth of *S. miltiorrhiza* plants and therefore it would be valuable to clarify the underlying molecular mechanism on how the above candidate TFs synchronously modulate the accumulation of medicinal substances and nitrogen signaling.

### *SmMYB53* positively regulates tanshinone biosynthesis but decreases phenolic acid accumulation in *S. miltiorrhiza* hairy roots

*SmMYB53* shared high nucleotide sequence homology with *SlMYB1* (Fig. 1B), which was validated to promote flavonoid accumulation in tomato [22]. This led us to explore the function of *SmMYB53* in modulating medicinal metabolite biosynthesis in *S. miltiorrhiza*. By genetic transformation, we confirm that *SmMYB53* positively regulates tanshinone biosynthesis. Thus, comparing the nucleotide sequence homology of candidate genes in different species before functional identification would be an efficient research strategy to deduce the role of candidate genes.

In *SmMYB53* transgenic hairy root lines, due to employing two different vectors in the *SmMYB53*-OE lines (*SmMYB53*-OE-11, -38, -67) and *SmMYB53*-RNAi lines (*SmMYB53*-RNAi-5, -21, -51), the DT, TI, and CT contents varied greatly in the two types of control transgenic hairy root lines with the vector pCAMBIA2306 in Fig. 2E and pFGC5941 in Fig. 2F, respectively. Actually, in overexpression, RNAi or CRISPR knockout genetic transformation experiments, metabolite contents in the control hairy root lines created by different transformation vectors exhibited an inconsistent trend [28]. In studying the SmAPK1-mediated phosphorylation of SmbZIP4 in positively regulating tanshinone biosynthesis in *S. miltiorrhiza*, the tanshinone contents, including DT, TI, CT, and TIIA, in two different wild types of *SmAPK1* overexpression and RNAi hairy root lines varied dramatically [29]. Therefore, we speculate that the control transgenic hairy roots employing different genetic transformation vectors may show an inconsistent physiological state that eventually leads to dramatic variations of metabolite contents in transgenic hairy roots.

In addition, the phenolic acid contents of *SmMYB53* transgenic hairy roots were measured by HPLC assay. Significant decreases in phenolic acids were observed in three *SmMYB53*-OE lines; in contrast, phenolic acid contents were increased in three *SmMYB53*-RNAi lines (Supplementary Data Fig. S6A and B). In *SmMYB53*-OE lines, the expression of *Sm4CL1*, *SmTAT1*, and *SmCYP98A14*, participating in the phenolic acid biosynthetic pathway, were all downregulated in comparison with the control, whereas the expression levels of the above three genes were upregulated in *SmMYB53*-RNAi lines (Supplementary Data Fig. S6C and D). Collectively, the results confirmed that *SmMYB53* positively regulates tanshinone biosynthesis but decreases phenolic acid accumulation in *S. miltiorrhiza* hairy roots.

### *SmMYB53* synchronously manipulates target genes and parallel genes involved in the tanshinone biosynthesis pathway

By genetic transformation experiments, we confirmed that *SmMYB53* promoted tanshinone accumulation in *SmMYB53*-OE hairy root lines (Fig. 2E) and decreased the tanshinone content in *SmMYB53*-RNAi hairy roots (Fig. 2F). Not only the downstream target *SmCYP71D375* gene but also three genes (*SmKSL1*, *SmGGPPS1*, and *SmCYP76AH1*) participating in the tanshinone biosynthesis pathway were all upregulated (Fig. 2G and H and Supplementary Data Fig. S4). The dual-LUC assay was used to further verify that *SmCYP71D375* was the only downstream target of *SmMYB53* (Fig. 4A and B). Overexpressing *SmERF1L1* upregulated five genes (*DXS*, *DXR*, *HMGS*, *CPS*, and *KSL*); however, *DXR* was the only target of *SmERF1L1* [14]. It was also demonstrated that *SmEIL1* activated *SmHMGR1* and *SmCPS1* expression, but *SmCPS1* alone was confirmed as the target of *SmEIL1* [30]. In conclusion, these findings imply that overexpressing certain TFs may manipulate not only the target genes but also the connected genes involved in the identical metabolic synthesis pathway. Therefore, overexpression of certain TFs would be a valuable strategy to improve the biosynthesis of bioactive substances in medicinal plants.

### *SmMYB53* might perform diverse biological functions in *S. miltiorrhiza*

With SmMYB53 as the bait, not only SmbZIP51 but also other TFs, including MYB1R1 (NCBI accession number XM_057918772.1), were captured. MYB TFs play key roles in regulating secondary metabolite biosynthesis in plants. Usually, MYB TFs recruit other TFs to form protein modules in the process of regulating plant secondary metabolism [31]. As reported previously, SmMYB1 was shown to interact with SmMYC2 to additively promote *CYP98A14* expression compared with the action of SmMYB1 alone, by which it improved the biosynthesis of phenolic acids in *S. miltiorrhiza* hairy roots [32]. TmMYB39 interacts with TmbHLH13 to regulate *GGPPS* and *T10OH* expression, thereby affecting paclitaxel biosynthesis in *Taxus media* [33]. In fruit, it has been validated that MdMYB305 competes with MdMYB10 to modulate the expression of the downstream *MdbHLH33* gene, subsequently balancing sugar and anthocyanin accumulation [34]. In the present study, we confirm that SmbZIP51 interacts with SmMYB53 to inhibit its activation of the expression of *SmCYP71D375*, subsequently regulating tanshinone biosynthesis in *S. miltiorrhiza* hairy roots. Moreover, several biotic- and abiotic-responsive genes were obtained by yeast library screening (Supplementary Data Table S3). The above findings imply that *SmMYB53* might perform diverse functions in *S. miltiorrhiza*.

### A proposed model for the role of *SmMYB53* in tanshinone biosynthesis

**Figure 5:**
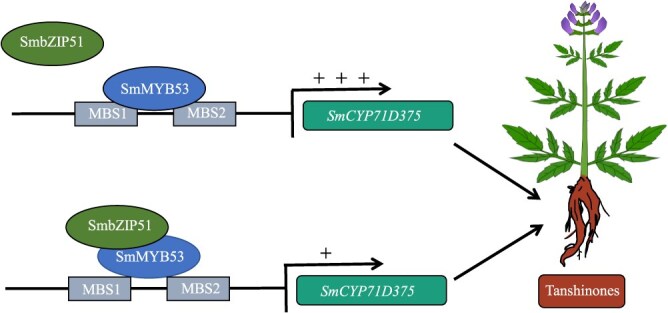
Proposed model for the role of SmMYB53 interaction with SmbZIP51 in modulating tanshinone biosynthesis in *S. miltiorrhiza*. *SmMYB53* activates the expression of *SmCYP71D375* to promote tanshinone biosynthesis by binding with the *cis*-elements (MBS1 and MBS2) present in *SmCYP71D375*. SmbZIP51 interacts with SmMYB53 to inhibit its activation of the expression of *SmCYP71D375*.

This study, to our knowledge, is the first report to identify the R2R3-MYB TF with the *SmCYP71D375* gene as the direct target, the downstream gene participating in tanshinone accumulation. As shown in the deduced model (Fig. 5), *SmMYB53* activates the expression of *SmCYP71D375*, the most downstream gene of the tanshinone biosynthesis pathway, by binding with the MBS1/2 *cis*-elements present in its promoter, thereby upregulating tanshinone biosynthesis. As a parallel regulator, SmbZIP51 interacts with SmMYB53 to inhibit its activation of the expression of *SmCYP71D375*.

*SmCYP71D375* is a newly identified downstream synthetase catalyzing hydroxylation to produce furan D-ring tanshinones from the precursor of phenolic abietane-type diterpenoids commonly existing in Lamiaceae plants, whereas the regulatory network with *SmCYP71D375* as the direct target modulated by candidate TFs is still unclear [13]. This study demonstrates the molecular mechanism of tanshinone accumulation modulated by *SmMYB53* with *SmCYP71D375* as the target gene, and offers a research basis for genetic improvement of medicinal component biosynthesis in *S. miltiorrhiza*.

## Materials and methods

### Plant materials

Different *S. miltiorrhiza* samples, including leaf, stem, root, and flower, used in this study were collected according to previously reported methods [32]. *Nicotiana benthamiana* seeds were sterilized and sown on MS medium (pH 5.8) in a tissue culture room for 12 d and then subcultivated in soil for 3 weeks for transient transformation by *Agrobacterium tumefaciens* [35]. Transgenic hairy roots were cultivated in ½ MS liquid medium with a rotation speed of 120 rpm under darkness at 25°C. All the tissues were collected and stored for RNA isolation [32].

### Construction of recombinant vector and generation of transgenic hairy roots

The ORF of *SmMYB53* was cloned from *S. miltiorrhiza* cDNA and inserted into the pCAMBIA2306-flag vector, acting as a recombinant overexpression vector (Supplementary Data Fig. S7A). For *SmMYB53-*RNAi vector construction, a unique fragment of *SmMYB53* (230–462 bp) was isolated and inserted into the NcoI and AscI sites of the pFGC5941 vector driven by the CaMV 35S promoter. This sequence was then inserted reversely into the BamHI and XbaI sites of the pFGC5941 vector (Supplementary Data Fig. S7B). Finally, the vectors constructed as described above were transferred into *Agrobacterium rhizogenes* C58C1, and then used to infect the injured explants to generate transgenic hairy roots [36]. Primers for constructing plasmids are listed in Supplementary Data Table S4. Genomic DNA of the transgenic hairy roots was extracted by the cetyltrimethylammonium bromide (CTAB) method [37]. Transgenic hairy roots were distinguished by PCR detection using 2 × Taq Master Mix (Vazyme, Nanjing, China) as described previously [32].

### Bioinformatics analysis of *SmMYB53*

A phylogenetic tree of AtMYB proteins and SmMYB53 was constructed by MEGA 6.0 software with the neighbor-joining (NJ) method. The reliability of the phylogenetic tree was evaluated by bootstrap analysis with 1000 replicates [38]. The protein sequences of AtMYB1, AtMYB25, AtMYB109, OsMYB1, SlMYB1, and SmMYB53 were aligned using DNAMAN 7.0 software [39].

### Subcellular localization

The ORF of *SmMYB53* was fused to PHB-35S::YFP vector to form the recombinant PHB-35S::*SmMYB53*-YFP, which was then transferred into *A. tumefaciens* GV3101. The PHB-35S::YFP vector was set as a negative control. The two recombinants were expressed transiently in transgenic *N. benthamiana* plants by the *A. tumefaciens* infiltration method as reported previously [35]. After 48 h of genetic transformation, the fluorescence signal in the nuclei of *N. benthamiana* mesophyll cells was observed after the injection of 4′6-diamidino-2-phenylindole dihydrochloride (DAPI) solution for 3 h with continuous cultivation prior to microscopic observation [35].

### RNA isolation and qRT–PCR detection

Total RNA of different tissues, including leaf, stem, root, flower, and hairy root, was extracted with an RNA extraction kit (Vazyme, Nanjing, China). For cDNA synthesis, about 2 μg RNA from each tissue was reverse-transcribed into cDNA using a reverse transcription kit (Vazyme, Nanjing, China) [36], and used as templates in qRT–PCR detection with specific primer pairs. The *SmActin* gene was set as the reference [40]. The comparative Ct method was used to evaluate the relative expression levels of candidate genes [41]. Each reaction was repeated three times.

### Extraction and quantitative determination of tanshinones

Transgenic hairy roots cultured continuously for 50 days were collected and ground to a powder with a pestle after freeze-drying with liquid nitrogen. For the extraction of tanshinones, methanol and dichloromethane were mixed at the ratio of 3:1. Hairy root powder (0.1 g) was placed in a 50-mL centrifuge tube and 16 mL of the mixed reagent was added, and the preparation was placed in the dark overnight. Later, centrifugation was performed at 7000 rpm for 10 min, and the supernatant was spin-steamed at 50°C. After spin-drying, the supernatant was redissolved with 1.5 mL methanol then passed through a filter membrane to conduct HPLC detection (Agilent, Palo Alto, CA, USA) as described previously [30].

### Yeast one-hybrid assay

The PlantCARE database was used to predict the MYB binding sites (MBS1 and MBS2) in the *SmCYP71D375* promoter. Then, MBS1 and MBS2 *cis*-elements were recombined into the pHIS vector. Each of the recombinant pHIS vectors was set as the bait. A complement of both the bait and the *S. miltiorrhiza* yeast library was co-transformed into the yeast strain Y187. After cultivation on SD/−Leu/−Trp/−His medium with the addition of 100 mM 3-AT for 48 h, the visible yeast monoclones were picked out, and each of them was used as template to amplify the inserted sequences within pGADT7 vector by the special primers shown in Supplementary Data Table S4. Finally, the PCR amplification fragments were subjected to sequencing to obtain *SmMYB53*. A Y1H experiment was used to further examine the activation of SmMYB53 binding to the *SmCYP71D375* promoter. The ORF of *SmMYB53* was amplified and recombined into plasmid pGADT7. Later, it was co-transformed with the above recombinant pHIS vector to determine whether SmMYB53 binds to MBS1 or MBS2. Plasmid pGADT7 was set as the negative control [42].

### Yeast two-hybrid assay

As described in a previous report [43], to capture the interactional protein of SmMYB53 by the Y2H assay, *SmMYB53* was recombined into the pGBKT7 plasmid as the bait, then the pGBKT7*-SmMYB53* construct and the *S. miltiorrhiza* yeast library (prey vector) were co-transformed into yeast strain AH109 and grown on SD/−Leu/−Trp/−His/−Ade medium (QDO) for 3 days. The well-grown monoclonal yeast spots were dipped as the template to clone the inserted gene within the pGADT7 vector, and then the PCR amplification sequences were subjected to sequencing for capturing SmbZIP51. To further verify the interaction between SmbZIP51 and SmMYB53, *SmbZIP51* was amplified and inserted into pGADT7 vector to form pGADT7-*SmbZIP51* (prey vector). The prey and bait (pGBKT7-*SmMYB53*) constructs were co-transformed into yeast strain AH109, which was then grown on SD/−Trp/−Leu medium (DDO) for 48 h. Candidate positive colonies were also further confirmed by PCR amplification and measured on QDO medium.

### Dual-luciferase assay

The *SmCYP71D375* promoter was amplified from the *S. miltiorrhiza* cDNA and recombined into the pGreenII 0800-LUC plasmid as a reporter construct, and then it was transformed into *A. tumefaciens* strain GV3101 with the helper plasmid of pSoup19. Recombinants of PHB-35S::*SmMYB53*-YFP and PHB-35S::*SmbZIP51*-YFP were individually constructed as the effectors, with PHB-35S::YFP as the negative control. *Agrobacterium* cells harboring the reporter construct and effector construct were re-suspended separately with the infiltration buffer and mixed in a ratio of 1:9 to adjust to a final OD_600_ of 0.75, and then transfected into sterile *N. benthamiana* mesophyll cells. Three days after infiltration, the enzyme activities of firefly luciferase (LUC) and *Renilla* luciferase (REN) were measured [28]. Each experiment was repeated three times.

### Bimolecular fluorescence complementation assay

The full-length ORF of *SmMYB53* was recombined into the pXY104 vector to form plasmid pXY104-*SmMYB53*-cYFP. Likewise, the ORF of *SmbZIP51* was inserted into the pXY106 vector to form plasmid pXY106-*SmbZIP51*-nYFP. All the recombinants were individually transformed into *A. tumefaciens* strain GV3101 as reported previously [44]. *Agrobacterium tumefaciens* cells containing *SmMYB53*-cYFP and *SmbZIP51*-nYFP were mixed in a ratio of 1:1, and then transfected into sterile *N. benthamiana* mesophyll cells with a syringe. Forty-eight hours after infiltration, the fluorescence signal was visualized by microscopic observation (Zeiss LSM880, Oberkochen, Germany). YFP signals and fluorescence excitation were detected at 488 nm and 515–545 nm, respectively [35].

### GST pull-down assay

The ORF of *SmMYB53* was inserted into the pCold plasmid in fusion with the His tag. Likewise, *SmbZIP51* was recombined into the pGEX4T-1 plasmid in fusion with the GST tag. All the recombinants were individually transformed into *Escherichia coli* strain BL21 to generate fusion protein with the induction of 0.5 mM isopropyl β-d-1-thiogalactopyranoside (IPTG), and were further purified by Ni-NTA resin and a GST-tag protein purification kit (Sangon, China) [45]. The purified SmbZIP51-GST protein was incubated with GST magnetic beads in equal volume at 25°C for 30 min to make the GST magnetic beads adsorb *SmbZIP51*-GST fusion protein adequately. Then, an equal volume of purified SmMYB53-His protein was added to the above reactions, kept at 4°C for more than 3 h. Then, the magnetic beads were washed three times with PBS. Finally, we collected the fusion proteins from the magnetic beads to conduct SDS–PAGE analysis and detection with anti-His and anti-GST antibody, and the empty GST tag was set as the negative control [44].

### Electrophoretic mobility shift assay

Two single-strand oligonucleotides containing MBS1 and MBS2 *cis*-elements respectively together with their flanking sequences were synthesized and labeled with biotin (Sunya, Hangzhou, China), and then annealed to double-stranded DNAs as in a previously reported method [46]. The labeled probes were individually incubated with the purified fusion proteins of *SmMYB53*-His and *SmbZIP51*-His in binding buffer for more than 30 min at 4°C [46]. The EMSA experiment was carried out using the LightShift Chemiluminescent EMSA Kit (Beyotime, China), and biotin signals were visualized on a chemiluminescence device (Tanon, China). Ten-fold amounts of the two unlabeled DNA fragments were set as competitors, respectively [27].

### Data statistics

All the detections performed in this study were repeated three times. The data obtained were visualized and statistically analyzed by GraphPad Prism 8 software. The significance of the data was evaluated by the *t*-test and one-way ANOVA.

## Supplementary Material

Web_Material_uhaf058

## Data Availability

All data generated in present study are included in this published article and its supplementary data files.
