# Supplementary material for: Molecular mechanism of SmMYB53 activates the expression of SmCYP71D375, thereby modulating tanshinone accumulation in Salvia miltiorrhiza
Source: Hortic Res. 2025 Feb 27;12(6):uhaf058. doi: 10.1093/hr/uhaf058 (PMC12017799; doi:10.1093/hr/uhaf058)
Supplement: Web_Material_uhaf058 [file web_material_uhaf058.zip › Supplementary Figure S2.pdf]

```
AGCCAGATGA TTAGTCTGAC AAATACTATG GGCTAATGCT TAAAGGAGCA CAACAAACCT ACCAATACCC
TCGGTCTACT AATCAGACTG TTTATGATAC CCGATTACGA ATTTCCTCGT GTTGTTTGGA TGGTTATGGG

TGGGTTGGAT TACTCGCTCG CTCTTAGCAC GTTAAGACAC ACTCGTGCCT TTGCCAGCGC TTGGCTAAGA
ACCCAACCTA ATGAGCGAGC GAGAATCGTG CAATTCTGTG TGAGCACGGA AACGGTCGCG AACCGATTCT

TATCTGGAGT CAGAACCCTG GTCTGAACTC CGCTCTACTC AAACACTATT TTCGCTCGGT TGAAAAAGGG
ATAGACCTCA GTCTTGGGAC CAGACTTGAG GCGAGATGAG TTTGTGATAA AAGCGAGCCA ACTTTTTCCC

TTTGAACTCT TCCAACTTAG ACTACTGAGA ACAGGCTCTC AAATAATCTA TCCTAGGTTT AAGATTAAAC
AAACTTGAGA AGGTTGAATC TGATGACTCT TGTCCGAGAG TTTATTAGAT AGGATCCAAA TTCTAATTTG

AGGGGTTGCC TAGAAATCTA AGAGAGTTTA TATAATCAGC TAGACTGATT TTACAACGTT TGAGTACTCT
TCCCCAACGG ATCTTTAGAT TCTCTAAAT ATATTAGTCG ATCTGACTAA AATGTTGCAA ACTCATGAGA

CTTCTTCAAT TCAAGGTTTT GTCTAAGCAA ATGCTGGCTT GTGATTTTGA CAGAGCTTCA GCGTATATCT
GAAGAAGTTA AGTTCCAAAA CAGATTCGTT TACGACCGAA CACTAAAACT GTCTCGAAGT CGCATATAGA

TGAATCGGTG AAGATTGCAG GCGTTCCTCA AGCTCTATTT ATAGGCGAAA TGTTGAATAG ATCCGTTGGA
ACTTAGCCAC TTCTAACGTC CGCAAGGAGT TCGAGATAAA TATCCGCTTT ACAACTTATC TAGGCAACCT

GAGATGATCT TCATGATTTC TGCCATTGTG AGATAAAGTC ACTTCTTGGG CTGAGGTGGC AATCTTCAAA
CTCTACTAGA AGTACTAAAG ACGGTAACAC TCTATTTCAG TGAAGAACCC GACTCCACCG TTAGAAGTTT

TGCTCCTTAT ACCAAAGGTT TGAATCGTCT TGTCCTTGGA GTATGGTGCT TCTGCATCTT TACACGCAAA
ACGAGGAATA TGGTTTCCAA ACTTAGCAGA ACAGGAACCT CATACCACGA AGACGTAGAA ATGTGCGTTT

GTGGAATTCT GGTACTTGCA AGGATGATCT TCAAATCTTC AGCATTAAAT GCACTTGTCC TTGAGCATTT
CACCTTAAGA CCATGAACGT TCCTACTAGA AGTTTAGAAG TCGTAATTTA CGTGAACAGG AACTCGTAAA

AATGCGGCGT GTCTGATACT CATTACTTTA ATTCAATGTA GAACATCGAC TGGTACTTGA TTTGAACTTC
TTACGCCGCA CAGACTATGA GTAATGAAAT TAAGTTACAT CTTGTAGCTG ACCATGAACT AAACTTGAAG

AGTTCGACCA TGACATGAAT TGTATGAGTA TCTCCGACTG GTGACTGATG GTTTTCAGTC AGTGTGCGTC
TCAAGCTGGT ACTGTACTTA ACATACTCAT AGAGGCTGAC CACTGACTAC CAAAAGTCAG TCACACGCAG

ATTCGGTAGT CCAATTCCGA AAGCTTCAGT TGAGCTGTCT TCAGTCTTCG GTCTTCAATA AGCTGGTCTT
TAAGCCATCA GGTTAAGGCT TTCGAAGTCA ACTCGACAGA AGTCAGAAGC CAGAAGTTAT TCGACCAGAA

CAGACAAAAG TCCTAATCTA AGCAGATGGA CACTAACAAT TGAGTCAGCG ACAAGACCTA TGCTAGAAGA
GTCTGTTTTC AGGATTAGAT TCGTCTACCT GTGATTGTTA ACTCAGTCGC TGTTCTGGAT ACGATCTTCT

TAAATTTAAG TGTTTTGGTA TCATCAAAAT TAGGATAAGG ATATTTCATT AATTTTTCCA ACAAGATGTT
ATTTAAATTC ACAAAACCAT AGTAGTTTTA ATCCTATTCC TATAAAGTAA TTAAAAAGGT TGTTCTACAA

TCTTTACTTA TATTTATTCT ATATAAAAAA ATTGAAATTG ATCAACTTAA CTAAAACGAC TAAAAAAATA
AGAAATGAAT ATAAATAAGA TATATTTTTT TAACTTTAAC TAGTTGAATT GATTTTGCTG ATTTTTTTAT

TTGATCAAAT TAATTAGAAC GGAAAAATTA TATTCATTTT TTTCCTTTTC AAATGCGGTA GTGTCCCATT
AACTAGTTTA ATTAATCTTG CCTTTTTAAT ATAAGTAAAA AAAGGAAAAG TTTACGCCAT CACAGGGTAA

TGTAACTTAG GATCAAATAA ATGCCTTAAA GTTTCACTTA GGAGTTAGGG GCGTATTTGG ACTTTATTCT
ACATTGAATC CTAGTTTATT TACGGAATTT CAAAGTGAAT CCTCAATCCC CGCATAAACC TGAAATAAGA

TTAAAGAAGG AGTTCTTTTA TATACGGGTA TCAAGTGAGA ATAACTATAT ACATATTTTA TGAGAAATAA
AATTTCTTCC TCAAGAAAAT ATATGCCCAT AGTTCACTCT TATTGATATA TGTATAAAAT ACTCTTTATT

GATTAACGAA TAAATTAATA CTCCCTCCGT CCACTAATTC AAGGCCTTCT TTCCTTTTTG GGACGTCCAC
CTAATTGCTT ATTTAATTAT GAGGGAGGCA GGTGATTAAG TTCCGGAAGA AAGGAAAAAC CCTGCAGGTG

CAACTCAAGG CCTATCCATT TTTAGTAAGT TTTTATTTAT ATTTAATTGG TGGGTCCCAA AACAATACAC
GTTGAGTTCC GGATAGGTAA AAATCATTCA AAAATAAATA TAAATTAACC ACCCAGGGTT TTGTTATGTG

TTTTTCTCCA CTCAACTAAT AAATAATACA TTAATTGTGG ATCCATTTCT CCACTCAACT AATAAAAATA
AAAAAGAGGT GAGTTGATTA TTTATTATGT AATTAACACC TAGGTAAAGA GGTGAGTTGA TTATTTTTAT

CATTTTTTCT TAAAACTCGT GTTTTGCTCC TTAGGTCATG AATTAGTGGA CGGATGGAGT AGAATTTAAT
GTAAAAAAGA ATTTTGAGCA CAAAACGAGG AATCCAGTAC TTAATCACCT GCCTACCTCA TCTTAAATTA

TATGAATAAC TTAATCTAAT ATATTAATAG TTGTATTCAG GTGTTACATT TATCTATTTG TGAATTTCAT
ATACTTATTG AATTAGATTA TATAATTATC AACATAAGTC CACAATGTAA ATAGATAAAC ACTTAAAGTA

CATATTAACA AATACTACTA TTCATATATT ATATGAATCT TATTAATTTA ATTATTATTT TCATTTTTTT
GTATAATTGT TTATGATGAT AAGTATATAA TATACTTAGA ATAATTAAAT TAATAATAAA AGTAAAAAAA

ACGAAAGATG GTCATTTTTT CTTCTAAACA TCTATCTATA TATGAATAAC ACACAACTTC TTCAACTTAG
TGCTTTCTAC CAGTAAAAAA GAAGATTTGT AGATAGATAT ATACTTATTG TGTGTTGAAG AAGTTGAATC

ACACGAGAAA TTTATACATT ATGGAGTTCA ACATCCCATC AACACTCATA GCTCTACTTT CATTTCTCCT
TGTGCTCTTT AAATATGTAA TACCTCAAGT TGTAGGGTAG TTGTGAGTAT CGAGATGAAA GTAAAGAGGA

CTTTCTCCTC ACGTTTCGCA AATCGCG
GAAAGAGGAG TGCAAAGCGT TTAGCGC
```

- A-box
- AAGAA-motif
- ABRE
- ARE
- AT~TATA-box
- Box 4
- CAAT-box
- CCAAT-box
- CCGTCC motif
- CCGTCC-box
- CGTCA-motif
- G-Box
- GA-motif
- GATA-motif
- GCN4_motif
- I-box
- LTR
- MBS
- MYB recognition site
- Myc
- STRE
- TCA
- TGA-element
- TGACG-motif
- WRE3
- as-1
- chs-CMA2a
- circadian
