# Supplementary material for: Molecular mechanism of SmMYB53 activates the expression of SmCYP71D375, thereby modulating tanshinone accumulation in Salvia miltiorrhiza
Source: Hortic Res. 2025 Feb 27;12(6):uhaf058. doi: 10.1093/hr/uhaf058 (PMC12017799; doi:10.1093/hr/uhaf058)
Supplement: Web_Material_uhaf058 [file web_material_uhaf058.zip › Supplementary Figure.docx]

**Molecular mechanism of *SmMYB53* activates the expression of *SmCYP71D375* thereby modulating the tanshinones accumulation in *Salvia miltiorrhiza***

Xinyu Wang^†^, Yifei Shi^†^, Qichao Wang, Xinjia Xie, Siqi Gui, Jiening Wu, Limei Zhao, Xiaowei Zou, Guoyin Kai*, Wei Zhou*

Laboratory for Core Technology of TCM Quality Improvement and Transformation, School of Pharmaceutical Sciences, School of Pharmacy and Academy of Chinese Medical Science, Zhejiang Chinese Medical University, Hangzhou 310053, China.

Authors:

Miss Xinyu Wang: wxinyu0504@163.com

Mr. Yifei Shi: 1498435899@qq.com

Mr. Qichao Wang: 925955014@qq.com

Miss Xinjia Xie: 1193357732@qq.com

Miss Siqi Gui: 1399759627@qq.com

Miss Jiening Wu: 1423654117@qq.com

Mrs. Limei Zhao: beckyzlm@zcmu.edu.cn

Mrs. Xiaowei Zou: zouxiaowei@zcmu.edu.cn

† These authors contributed equally to this work.

* Corresponding authors:

Prof. Wei Zhou: 20171069@zcmu.edu.cn

Prof. Guoyin Kai: kaiguoyin@zcmu.edu.cn

**Running title:** *SmMYB53* activates the *SmCYP71D375* expression to regulate the tanshinones accumulation in *S. miltiorrhiza*.

**Supplementary data**

**
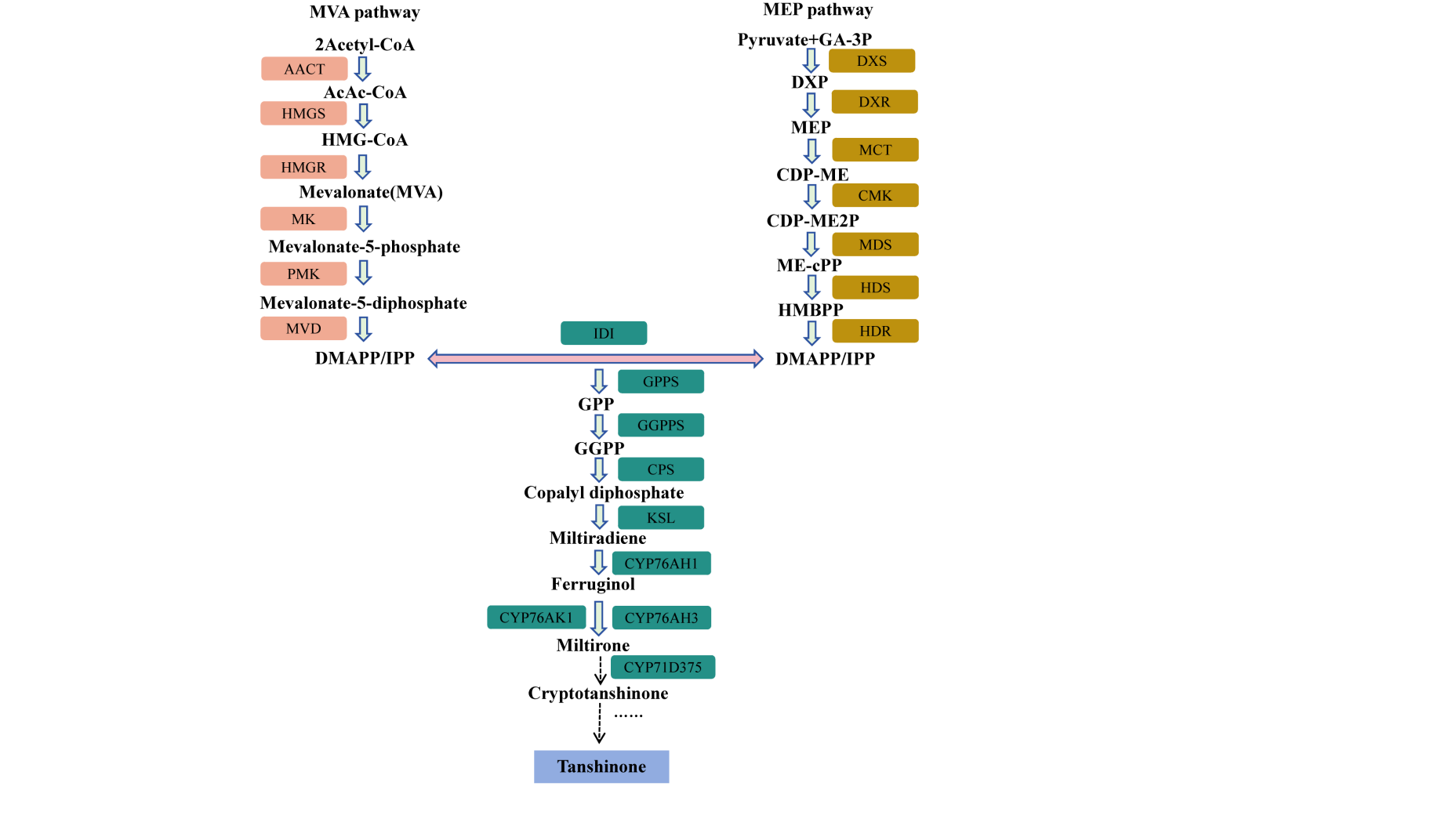
**

**Supplementary Figure S1.** Tanshinones biosynthesis pathway. Solid line represents the parsed catalytic steps and dotted line represents the unparsed catalytic steps.

**
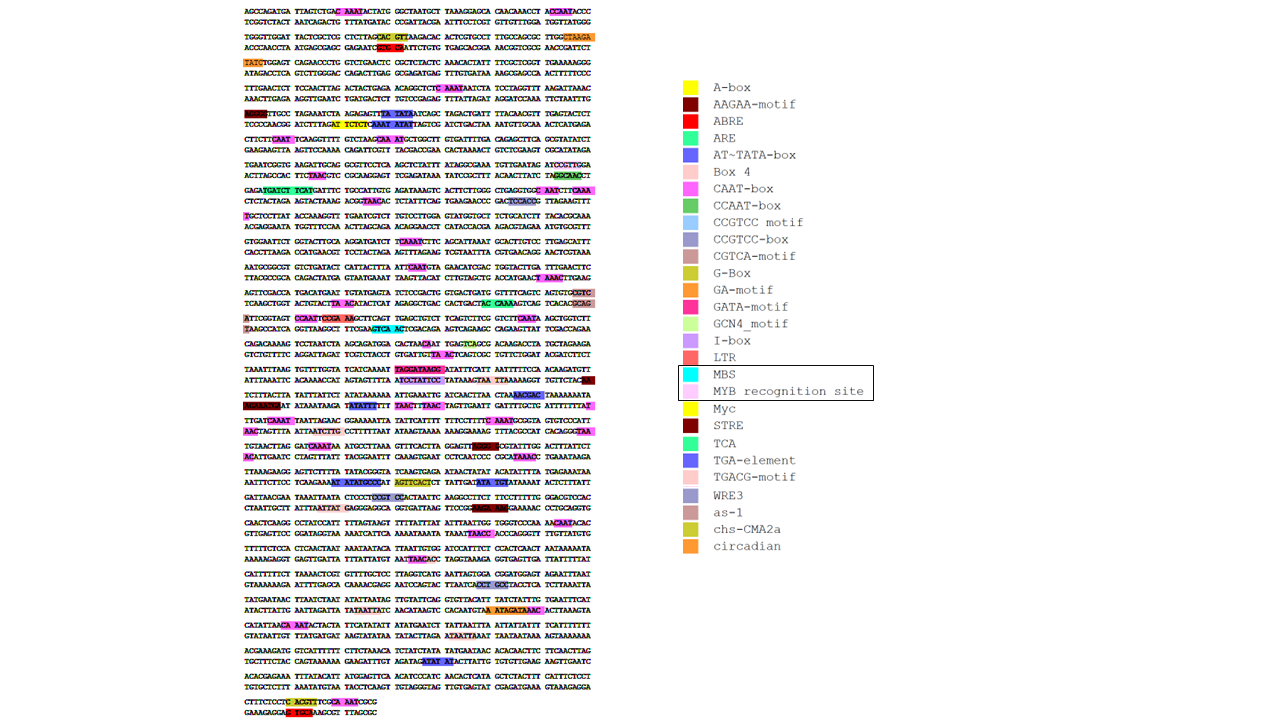
**

**Supplementary Figure S2.** C*is*-elements present in the *SmCYP71D375* promoter.


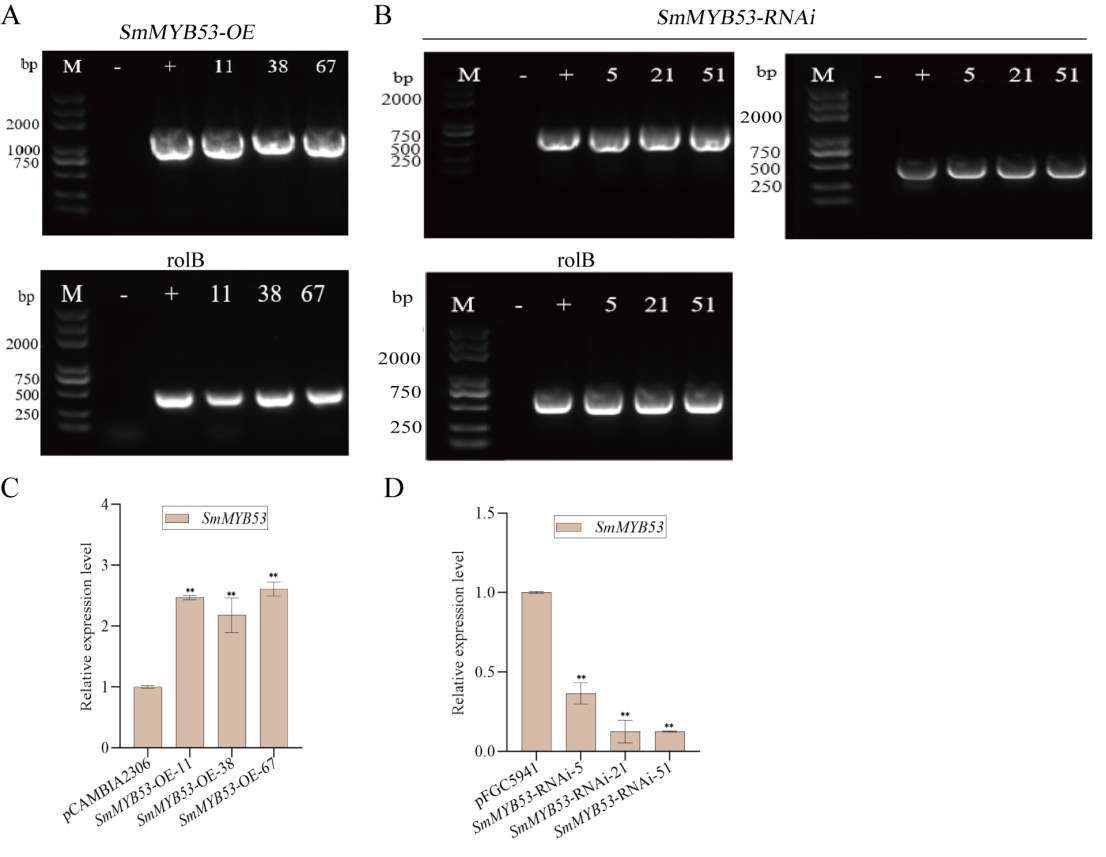


**Supplementary Figure S3.** Identification of *SmMYB53* transgenic hairy roots. (A, B) Identification of *SmMYB53*-OE (A) and *SmMYB53*-RNAi (B) transgenic hairy root lines by PCR. (M: marker; +: positive control; -: negative control). (C, D) Relative expression level of *SmMYB53* in *SmMYB53*-OE (C) and *SmMYB53-*RNAi (D) transgenic hairy roots. *SmActin* gene was used as the reference*.* Asterisks indicate significant differences by *t*-test (***p* < 0.01). Data are means of three biological replicates with the mean ± SD.


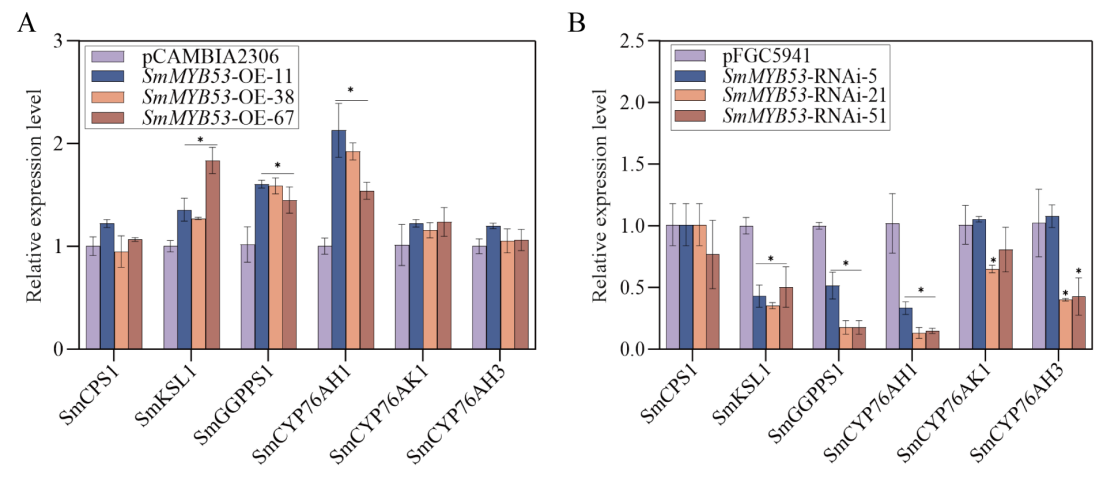
 **Supplementary Figure S4.** Relative expression level of genes participating in tanshinones accumulation pathway in *SmMYB53* transgenic hairy roots. (A) *SmMYB53*-OE transgenic hairy root lines. (B) *SmMYB53*-RNAi transgenic hairy root lines. *SmActin* gene was used as the reference*.* Asterisks indicate significant differences by *t*-test (**p* < 0.05). Data are means of three biological replicates with the mean ± SD.


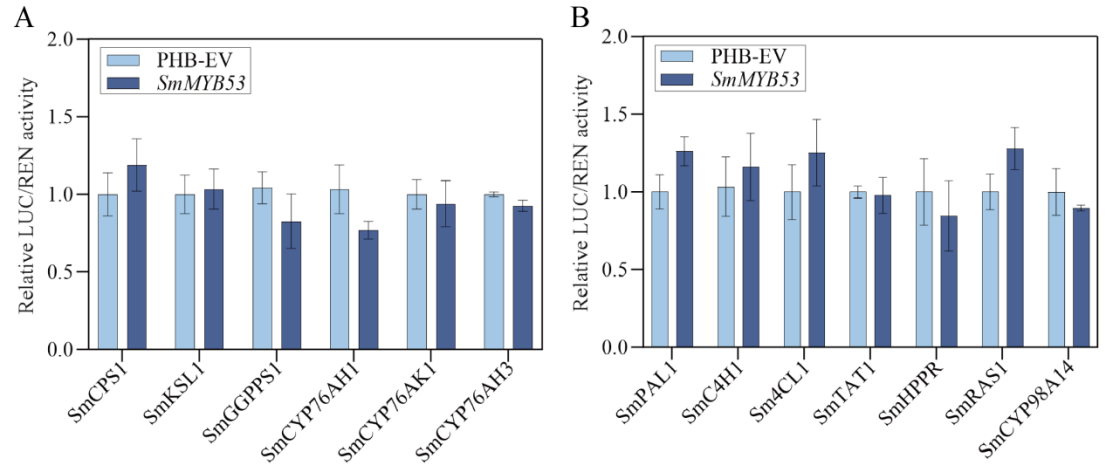


**Supplementary Figure S5**. Validation of SmMYB53 activating the promoter of genes participating in tanshinones and phenolic acids biosynthetic pathway by dual-LUC assays. LUC activities driven by the promoters of candidate genes (*PAL1, C4H1, 4CL1, TAT1, HPPR, RAS1, CYP98A14, CPS1, KSL1, GGPPS1, CYP76AH1, CYP76AK1* and *CYP76AH3*), respectively, were used as the reporter constructs. Effector vector not carrying *SmMYB53* was used as negative control (PHB-EV). Fold changes of the relative LUC activity were normalized to the activity of the control. All the detections were biologically replicated three times.


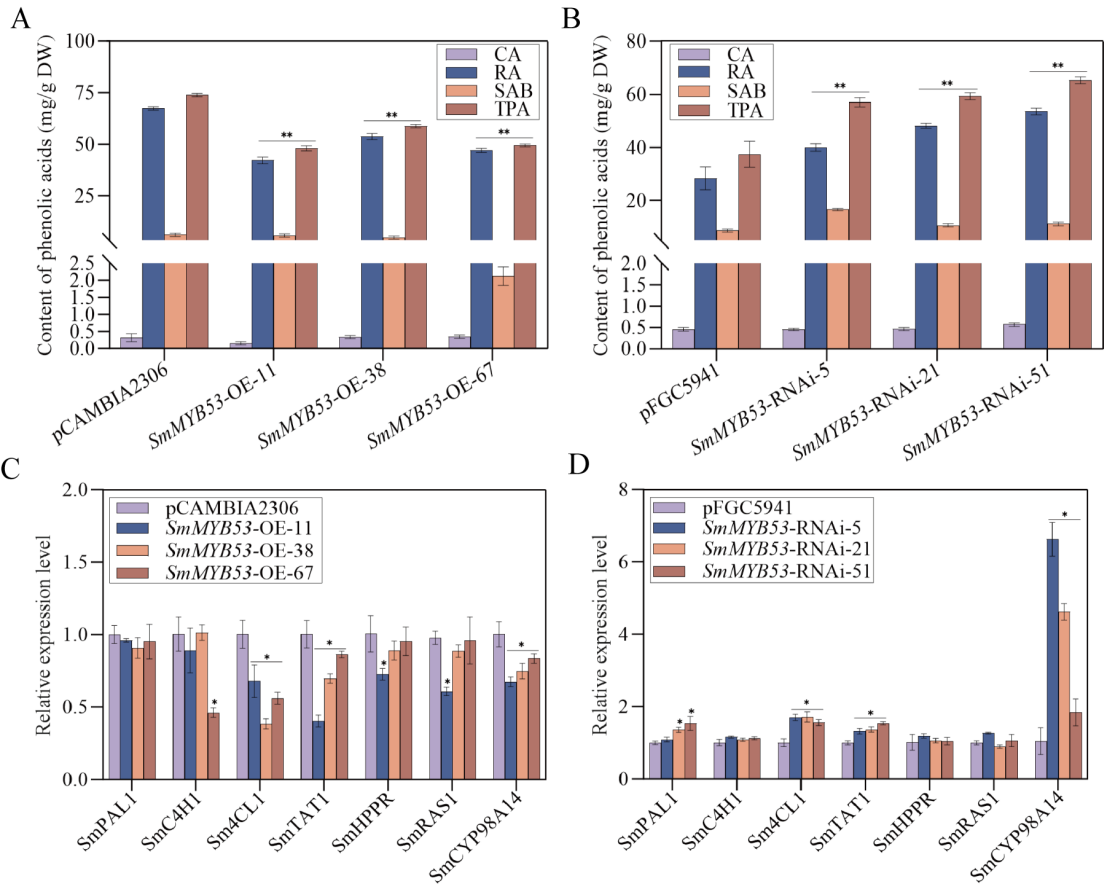


**Supplementary Figure S6**. Measuring the phenolic acids content and the relative expression level of genes participating in phenolic acids accumulation pathway in *SmMYB53* transgenic hairy roots. (A, B) Content of phenolic acids in *SmMYB53-*OE (A) and *SmMYB53-*RNAi (B) transgenic hairy root lines. (CA, caffeic acid; RA, rosmarinic acid; SAB, salvianolic acid B; TPA, total of phenolic acids). (C, D) Relative expression levels of genes involved in phenolic acids biosynthesis pathway in *SmMYB53-*OE (C) and *SmMYB53-*RNAi (D) transgenic hairy root lines. *SmActin* gene was used as the reference*.* Asterisks indicate significant differences by *t*-test (**p* < 0.05). Data are means of three biological replicates with the mean ± SD.


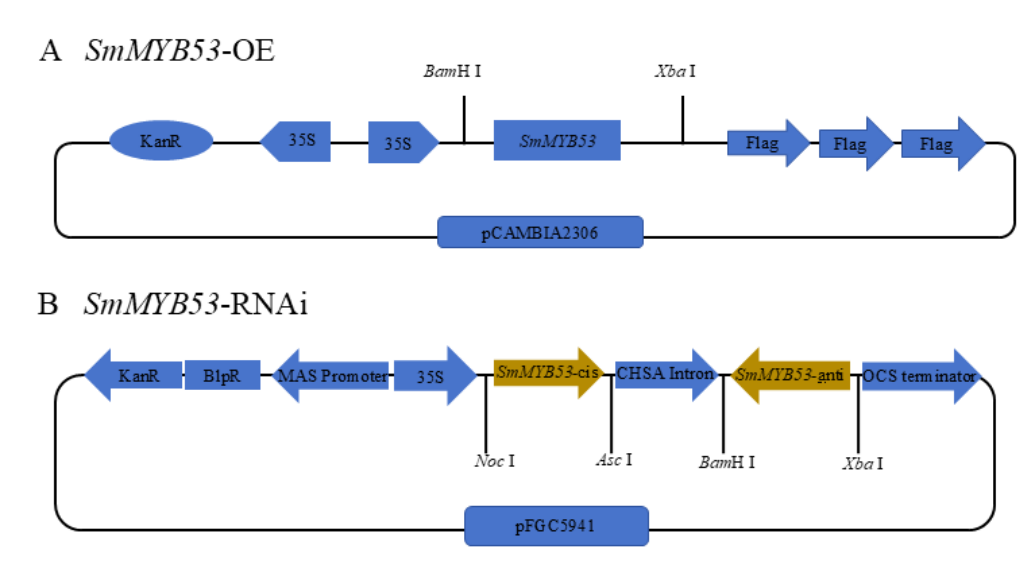


**Supplementary Figure S7.** Construction of the *SmMYB53*-OE and *SmMYB53-*RNAi recombinant vectors. (A) *SmMYB53*-OE recombinant plasmid for gene overexpression. (B) Construction of the *SmMYB53-*RNAi expression vector.
