# Supplementary material for: Molecular mechanism of SmMYB53 activates the expression of SmCYP71D375, thereby modulating tanshinone accumulation in Salvia miltiorrhiza
Source: Hortic Res. 2025 Feb 27;12(6):uhaf058. doi: 10.1093/hr/uhaf058 (PMC12017799; doi:10.1093/hr/uhaf058)
Supplement: Web_Material_uhaf058 [file web_material_uhaf058.zip › Supplementary Table(1).docx]

**Molecular mechanism of *SmMYB53* activates the expression of *SmCYP71D375* thereby modulating the tanshinones accumulation in *Salvia miltiorrhiza***

Xinyu Wang^†^, Yifei Shi^†^, Qichao Wang, Xinjia Xie, Siqi Gui, Jiening Wu, Limei Zhao, Xiaowei Zou, Guoyin Kai*, Wei Zhou*

Laboratory for Core Technology of TCM Quality Improvement and Transformation, School of Pharmaceutical Sciences, School of Pharmacy and Academy of Chinese Medical Science, Zhejiang Chinese Medical University, Hangzhou 310053, China.

Authors:

Miss Xinyu Wang: wxinyu0504@163.com

Mr. Yifei Shi: 1498435899@qq.com

Mr. Qichao Wang: 925955014@qq.com

Miss Xinjia Xie: 1193357732@qq.com

Miss Siqi Gui: 1399759627@qq.com

Miss Jiening Wu: 1423654117@qq.com

Mrs. Limei Zhao: beckyzlm@zcmu.edu.cn

Mrs. Xiaowei Zou: zouxiaowei@zcmu.edu.cn

† These authors contributed equally to this work.

* Corresponding authors:

Prof. Wei Zhou: 20171069@zcmu.edu.cn

Prof. Guoyin Kai: kaiguoyin@zcmu.edu.cn

**Running title:** *SmMYB53* activates the *SmCYP71D375* expression to regulate the tanshinones accumulation in *S. miltiorrhiza.*

**Supplementary Table S1.** Mining of the preys with MBS1 *cis*-element as the bait by Y1H yeast library screening.

| Number | Gene ID | Frequency | Annotation |
| --- | --- | --- | --- |
| 3 | XM_057916772.1 | 1/100 | *Salvia miltiorrhiza* aromatic aminotransferase ISS1 (LOC130992222), mRNA |
| 10 | XM_057939264.1 | 1/100 | *Salvia miltiorrhiza* 30S ribosomal protein S10, chloroplastic-like (LOC131011469), mRNA |
| 15 | XM_057919695.1 | 1/100 | *Salvia miltiorrhiza* protein STAY-GREEN homolog, chloroplastic-like |
| 20 | XM_057908903.1 | 1/100 | *Salvia miltiorrhiza* peroxidase 42 (LOC130985786), mRNA |
| 24 | XM_057945137.1 | 1/100 | *Salvia miltiorrhiza* protein NLP6-like (LOC131016445), transcript variant X3, |
| 42 | XM_057936348.1 | 1/100 | *Salvia miltiorrhiza* ATP-dependent zinc metalloprotease FTSH 2, chloroplastic-like |
| 45 | KF059407.1 | 1/100 | *Salvia miltiorrhiza* MYB-related transcription factor (MYB53) mRNA, |
| 49 | XM_057927644.1 | 1/100 | *Salvia miltiorrhiza* F-box protein At2g02240-like (LOC131001291), mRNA |
| 50 | XM_057932713.1 | 1/100 | *Salvia miltiorrhiza* probable aquaporin PIP1-4 (LOC131005682), mRNA |
| 52 | XR_009096397.1 | 1/100 | *Salvia miltiorrhiza* phosphatidylglycerophosphate phosphatase 1, |
| 68 | XM_057945391.1 | 1/100 | *Salvia miltiorrhiza* heat shock cognate 70 kDa protein 2 (LOC131016687) |
| 70 | XM_057916973.1 | 1/100 | *Salvia miltiorrhiza* probable 2-oxoglutarate-dependent dioxygenase AOP1 (LOC130992373), transcript variant X2, mRNA |
| 72 | XM_057931980.1 | 1/100 | *Salvia miltiorrhiza* nicotinamidase 1-like (LOC131005162), mRNA |
| 79 | XM_057938035.1 | 1/100 | *Salvia miltiorrhiza* proteasome subunit alpha type-1-B-like (LOC131010500), mRNA |
| 87 | XM_057920165.1 | 1/100 | *Salvia miltiorrhiza* ubiquitin-conjugating enzyme E2 4 (LOC130994955), mRNA |
| 90 | XM_057910590.1 | 1/100 | *Salvia miltiorrhiza* mitogen-activated protein kinase kinase 2-like (LOC130987027), transcript variant X1, mRNA |
| 92 | XM_057917245.1 | 1/100 | *Salvia miltiorrhiza* YTH domain-containing protein ECT2-like (LOC130992563), mRNA |
| 96 | XM_057937652.1 | 1/100 | *Salvia miltiorrhiza* protein NRT1/ PTR FAMILY 5.10-like (LOC131010227), transcript variant X3, mRNA |
| 97 | XM_057948889.1 | 1/100 | *Salvia miltiorrhiza* pyruvate dehydrogenase E1 component subunit beta-1, mitochondrial-like (LOC131020196), transcript variant X2, mRNA |
| 100 | XM_057920165.1 | 1/100 | *Salvia miltiorrhiza* ubiquitin-conjugating enzyme E2 4 (LOC130994955), mRNA |

**Supplementary Table S2.** Mining of the preys with MBS2 *cis*-element as the bait by Y1H yeast library screening.

| Number | Gene ID | Frequency | Annotation |
| --- | --- | --- | --- |
| 4 | XM_057938812.1 | 1/100 | *Salvia miltiorrhiza* protein GET1-like (LOC131011059), transcript variant X5, mRNA |
| 10 | XM_057956461.1 | 1/100 | *Salvia miltiorrhiza* lectin-like (LOC131026572), transcript variant X3, mRNA |
| 18 | XM_057956450.1 | 1/100 | *Salvia miltiorrhiza* mannose/glucose-specific lectin Cramoll-like (LOC131026562), mRNA |
| 24 | XM_057916973.1 | 1/100 | *Salvia miltiorrhiza* probable 2-oxoglutarate-dependent dioxygenase AOP1 (LOC130992373), transcript variant X2, mRNA |
| 36 | EF593952.1 | 1/100 | *Salvia miltiorrhiza* SMLII mRNA, complete cds |
| 48 | XM_042181104.1 | 1/100 | *Salvia splendens* putative tRNA (cytidine(34)-2'-O)- methyltransferase (LOC121783060), transcript variant X1, mRNA |
| 56 | XM_048112333.1 | 1/100 | *Salvia hispanica* mediator of RNA polymerase II transcription subunit 10b-like (LOC125212232), mRNA |
| 60 | KF059407.1 | 1/100 | *Salvia miltiorrhiza* MYB-related transcription factor (MYB53) mRNA |
| 68 | XM_057909502.1 | 1/100 | *Salvia miltiorrhiza* zinc transporter 2 (LOC130986183), mRNA |
| 89 | XM_057918142.1 | 1/100 | *Salvia miltiorrhiza* 40S ribosomal protein S4-1-like (LOC130993305), mRNA |
| 90 | XM_057913258.1 | 1/100 | *Salvia miltiorrhiza* probable aquaporin PIP2-8 (LOC130989296), mRNA |
| 94 | XM_057952347.1 | 1/100 | *Salvia miltiorrhiza* nitrate regulatory gene2 protein-like (LOC131022822), mRNA |
| 98 | XM_057907891.1 | 1/100 | *Salvia miltiorrhiza* nuclear transcription factor Y subunit A-7-like (LOC130985112), transcript variant X3, mRNA |

**Supplementary Table S3.** Capturing the interactional proteins with SmMYB53 as the bait by Y2H yeast library screening.

| Number | Gene ID | Frequency | Annotation |
| --- | --- | --- | --- |
| 1 | XM_057920984.1 | 1/100 | *Salvia miltiorrhiza* chlorophyll a-b binding protein 7, chloroplastic-like (LOC130995627), mRNA |
| 5 | XM_057917084.1 | 1/100 | *Salvia miltiorrhiza* uncharacterized LOC130992448 (LOC130992448), transcript variant X2, mRNA |
| 6 | XM_042197847.1 | 1/100 | *Salvia splendens* zinc finger A20 and AN1 domain-containing stress-associated protein 6-like (LOC121798708), mRNA |
| 7 | XM_057939629.1 | 1/100 | *Salvia miltiorrhiza* ACT domain-containing protein ACR3 (LOC131011789), transcript variant X4, mRNA |
| 8 | XM_057956452.1 | 1/100 | *Salvia miltiorrhiza* lectin alpha chain-like (LOC131026564), mRNA |
| 9 | MK183051.1 | 1/100 | *Salvia miltiorrhiza* laccase 11 mRNA, complete cds |
| 10 | XM_057911535.1 | 1/100 | *Salvia miltiorrhiza* heavy metal-associated isoprenylated plant protein 7-like (LOC130987837), transcript variant X2, mRNA |
| 11 | XM_057939798.1 | 1/100 | *Salvia miltiorrhiza* increased DNA methylation 1-like (LOC131011899), transcript variant X1, mRNA |
| 13 | XM_057913428.1 | 1/100 | *Salvia miltiorrhiza* carbonic anhydrase 2-like (LOC130989425), mRNA |
| 14 | XM_057929848.1 | 1/100 | *Salvia miltiorrhiza* protein P21-like (LOC131003326), mRNA |
| 15 | XM_057931220.1 | 1/100 | *Salvia miltiorrhiza* F-box protein SKIP24 (LOC131004515), mRNA |
| 21 | XM_057943069.1 | 1/100 | *Salvia miltiorrhiza* patatin-like protein 6 (LOC131014911), transcript variant X1, mRNA |
| 33 | XM_057908069.1 | 1/100 | *Salvia miltiorrhiza* probable starch synthase 4, chloroplastic/amyloplastic (LOC130985229), transcript variant X2, mRNA |
| 35 | XM_057942986.1 | 1/100 | *Salvia miltiorrhiza* mRNA-decapping enzyme subunit 2-like (LOC131014859), mRNA |
| 47 | XM_057918772.1 | 1/100 | *Salvia miltiorrhiza* transcription factor MYB1R1 (LOC130993731), mRNA |
| 48 | XM_057914322.1 | 1/100 | *Salvia miltiorrhiza* zinc finger A20 and AN1 domain-containing stress-associated protein 8-like (LOC130990120), transcript variant X1, mRNA |
| 49 | XM_057929848.1 | 1/100 | *Salvia miltiorrhiza* protein P21-like (LOC131003326), mRNA |
| 50 | XM_057938104.1 | 1/100 | *Salvia miltiorrhiza* phenylcoumaran benzylic ether reductase TP7-like (LOC131010547), mRNA |
| 51 | XM_057939512.1 | 3/100 | *Salvia miltiorrhiza* transcription factor SmbZIP51 (LOC131011713), mRNA |
| 52 | XM_057927684.1 | 1/100 | *Salvia miltiorrhiza* glycine-rich RNA-binding protein 4, mitochondrial (LOC131001322), mRNA |
| 54 | XM_057954648.1 | 1/100 | *Salvia miltiorrhiza* 14 kDa proline-rich protein DC2.15-like (LOC131025051), mRNA |
| 55 | XM_057931361.1 | 1/100 | *Salvia miltiorrhiza* AAA-ATPase ASD, mitochondrial-like (LOC131004656), mRNA |
| 56 | XM_057920197.1 | 1/100 | *Salvia miltiorrhiza* catalase isozyme 2-like (LOC130995012), mRNA |
| 57 | XM_057920670.1 | 1/100 | *Salvia miltiorrhiza* uncharacterized LOC130995399 (LOC130995399), mRNA |
| 60 | XM_057913258.1 | 1/100 | *Salvia miltiorrhiza* probable aquaporin PIP2-8 (LOC130989296), mRNA |
| 62 | XM_057918289.1 | 1/100 | *Salvia miltiorrhiza* hevamine-A-like (LOC130993410), mRNA |
| 64 | XM_057926208.1 | 1/100 | *Salvia miltiorrhiza* uncharacterized LOC131000341 (LOC131000341), mRNA |
| 65 | XM_057910066.1 | 1/100 | *Salvia miltiorrhiza* cysteine proteinase 15A-like (LOC130986611), mRNA |
| 66 | XM_057913626.1 | 1/100 | *Salvia miltiorrhiza* early nodulin-like protein 17 (LOC130989612), mRNA |
| 67 | XM_057908924.1 | 1/100 | *Salvia miltiorrhiza* lactoylglutathione lyase GLX1 (LOC130985802), transcript variant X2, mRNA |
| 85 | XM_057947880.1 | 1/100 | *Salvia miltiorrhiza* glyoxylate/hydroxypyruvate reductase HPR3-like (LOC131019274), mRNA |
| 89 | XM_057936308.1 | 1/100 | *Salvia miltiorrhiza* heat shock 22 kDa protein, chloroplastic-like (LOC131009095), transcript variant X1, mRNA |
| 90 | XM_057949039.1 | 1/100 | *Salvia miltiorrhiza* ribulose bisphosphate carboxylase small subunit, chloroplastic-like (LOC131020308), mRNA |
| 91 | XM_057934426.1 | 1/100 | *Salvia miltiorrhiza* 60S ribosomal protein L18-2 (LOC131007279), mRNA |
| 96 | XM_057946007.1 | 1/100 | *Salvia miltiorrhiza* uncharacterized LOC131017282 (LOC131017282), mRNA |
| 98 | XM_057939287.1 | 1/100 | *Salvia miltiorrhiza* 40S ribosomal protein S8-like (LOC131011508), mRNA |

**Supplementary Table S4.** List of primers.

| Primer names | Primer sequences (5’-3’) | Notes |
| --- | --- | --- |
| T7-F | TAATACGACTCACTATAGG | Amplifying the inserted genes in the pGADT7 vector |
| 3’AD-R | AGATGGTGCACGATGCACAG |  |
| pGREEN0800-PstI-  *SmCYP71D375pro*-F | AAGCTTGATATCGAATTCCTGCAGCAGCCAGATGATTAGTCTGACAAATAC | Cloning the *SmCYP71D375* promoter |
| pGREEN0800-BamHI-  *SmCYP71D375pro*-R | GGCCGCTCTAGAACTAGTGGATCCTCTCGTGTCTAAGTTGAAGAAGTTGTG |  |
| pGEX4T-1-*SmbZIP51*-EcoRI-F | CGGATCCCCGGAATTCATGATGGACGTCGACCCCA | Amplifying the ORF of *SmbZIP51* and *SmMYB53* to express the proteins |
| pGEX4T-1-*SmbZIP51*-XhoI-R | ATGCGGCCGCTCGAGTGGACTGCGTCTCGCGT |  |
| pCold-*SmMYB53*-NdeI-F | GGTATCGAAGGTAGGCATATGATGGAGATCGATACTCAAGCAGCG |  |
| pCold-*SmMYB53*-SalI-R | CTATCTAGACTGCAGGTCGACCATCGTCTCCAAAATGCCTCGTT |  |
| pXY104-*SmbZIP51*-BamHI-F | ATTACAGGTACCCGGGGATCCATGATGGACGTCGACCCCA | BiFC assay |
| pXY104-*SmbZIP51*-XbaI-R | GCCACCGCCGTCGACTCTAGATGGACTGCGTCTCGCGT |  |
| pXY106-*SmMYB53*-BamHI-F | ATCGAGGACGCCGGCGGATCCATGGAGATCGATACTCAAGCAGCG |  |
| pXY106-*SmMYB53*-XbaI-R | GCTCTGCAGGTCGACTCTAGACATCGTCTCCAAAATGCCTCGTT |  |
| pCAMBIA2306-*SmMYB53*-BamHI-F | ACGAGCTCGGTACCCGGGGATCCATGGAGATCGATACTCAAGCAGCG | Construction of *SmMYB53* overexpression vector |
| pCAMBIA2306-*SmMYB53*-XbaI-R | AGGGCGAATTGGTCGACTCTAGACATCGTCTCCAAAATGCCTCGTT |  |
| pFGC5941-*SmMYB53*-NcoI-F | TTACATTTACAATTACCATGGGTGGAATTCCCGGCAGATCG | Construction of *SmMYB53-*RNAi vector |
| pFGC5941-*SmMYB53*-AscI-R | TTAAATCATCGATTGGGCGCGCCCCAAGCTCCAAGCAACGACG |  |
| pFGC5941-*SmMYB53*-XbaI-F | GGTCTTAATTAACTCTCTAGAGTGGAATTCCCGGCAGATCG |  |
| pFGC5941-*SmMYB53*-BamHI-R | AATTTGCAGGTATTTGGATCCCCAAGCTCCAAGCAACGACG |  |
| pGADT7-*SmbZIP51*-EcoRI-F | TGGCCATGGAGGCCAGTGAATTCATGATGGACGTCGACCCCA | Y1H assay |
| pGADT7-*SmbZIP51*-BamHI-R | TGCAGCTCGAGCTCGATGGATCCTGGACTGCGTCTCGCGT |  |
| pGBKT7-*SmMYB53*-EcoRI-F | ATGGCCATGGAGGCCGAATTCATGGAGATCGATACTCAAGCAGCG |  |
| pGBKT7-*SmMYB53*-PstI-R | CTAGTTATGCGGCCGCTGCAGCATCGTCTCCAAAATGCCTCGTT |  |
| pHIS-*SmCYP71D375pro*-MBS2-F | AATTCCCGTTGCCGTTGCCGTTGGAGCT |  |
| pHIS-*SmCYP71D375pro*-MBS2-R | CCAACGGCAACGGCAACGGG |  |
| pHIS-*SmCYP71D375pro*-MBS1-F | AATTCCAGTTGCAGTTGCAGTTGGAGCT |  |
| pHIS-*SmCYP71D375pro*-MBS1-R | CCAACTGCAACTGCAACTGG |  |
| EMSA-*SmCYP71D375pro*-MBS2-F | AGATCCGTTGGAGAAGATCCGTTGGAGAAGATCCGTTGGAGA | EMSA assay |
| EMSA-*SmCYP71D375pro*-MBS2-R | TCTCCAACGGATCTTCTCCAACGGATCTTCTCCAACGGATCT |  |
| EMSA-Mutant-*SmCYP71D375pro*-MBS2-F | AGATAAAAAAGAGAAGATAAAAAAGAGAAGATAAAAAAGAGA |  |
| EMSA-Mutant-*SmCYP71D375pro*-MBS2-R | TCTCTTTTTTATCTTCTCTTTTTTATCTTCTCTTTTTTATCT |  |
| EMSA-Mutant-*SmCYP71D375pro*-MBS1-F | GCTTAAAAAAAGCTGCTTAAAAAAAGCTGCTTAAAAAAAGCT |  |
| EMSA-Mutant-*SmCYP71D375pro*-MBS1-R | AGCTTTTTTTAAGCAGCTTTTTTTAAGCAGCTTTTTTTAAGC |  |
| EMSA-*SmCYP71D375pro*-MBS1-F | GCTTCAGTTGAGCTGCTTCAGTTGAGCTGCTTCAGTTGAGCT |  |
| EMSA-*SmCYP71D375pro*-MBS1-R | AGCTCAACTGAAGCAGCTCAACTGAAGCAGCTCAACTGAAGC |  |
| PHB-SmbZIP51-BamHI-F | CTCTCTCTCAAGCTTGGATCCATGATGGACGTCGACCCCA | dual-LUC assay |
| PHB-*SmbZIP51*-SpeI-R | GCCCTTGCTCACCATACTAGTTGGACTGCGTCTCGCGT |  |
| PHB-*SmMYB53*-BamHI-F | CTCTCTCTCAAGCTTGGATCCATGGAGATCGATACTCAAGCAGCG |  |
| PHB-*SmMYB53*-SpeI-R | GCCCTTGCTCACCATACTAGTCATCGTCTCCAAAATGCCTCGTT |  |
| qRT-PCR-*SmCYP76AH1*-F | TGCCCAACTTCGCCGACTACTTC | Quantitative real time PCR analysis |
| qRT-PCR-*SmCYP76AH1*-R | GTGTTCGTGTCCGATCCTCCCAC |  |
| qRT-PCR-*SmCPS1*-F | GATCGCCTCGTCAATACCAT |  |
| qRT-PCR-*SmCPS1*-R | TTCGAACCCACAAGTCATGT |  |
| qRT-PCR-*SmKSL1*-F | GTGTGACCCTTCTGCTAGCA |  |
| qRT-PCR-*SmKSL1*-R | TGCATTGTCTTGGGAAGATG |  |
| qRT-PCR-*SmGGPPS1*-F | GCTGTGCTCGCAGGGGATG |  |
| qRT-PCR-*SmGGPPS1*-R | ATCGCCGGTGCAGTTCAGG |  |
| qRT-PCR-*SmCYP76AH3*-F | CAAGTCGTCAACGGCTACCTCATC |  |
| qRT-PCR-*SmCYP76AH3*-R | GGTTGGAAGGCGTCTGGATTGTTC |  |
| qRT-PCR-*SmCYP76AK1*-F | CAAGCACGAGACTCCTCCGATTAC |  |
| qRT-PCR-*SmCYP76AK1*-R | GGTGTCCGATCCTGCGATGATTATG |  |
| qRT-PCR-*SmCYP71D375*-F | AGTGAATGCATGGGCACTGG |  |
| qRT-PCR-*SmCYP71D375*-R | AGCATAGCAAGCGGAAGCTC |  |
| qRT-PCR-*SmTAT1*-F | CAACTGCTGGTCTTCCACAAAC |  |
| qRT-PCR-*SmTAT1*-R | GCGAGCCAAAACGGACA |  |
| qRT-PCR-*SmPAL1*-F | GATAGCGGAGTGCAGGTCGTAC |  |
| qRT-PCR-*SmPAL1*-R | CGAACTAGCAGATTGGCAGAGG |  |
| qRT-PCR-*SmC4H1*-F | CCAGGAGTCCAAATAACAGAGC |  |
| qRT-PCR-*SmC4H1*-R | GCCACCAAGCGTTCACCAAGAT |  |
| qRT-PCR-*Sm4CL1*-F | CAGCAAGTGGACGGCGAGAATC |  |
| qRT-PCR-*Sm4CL1*-R | GACACGCAGACAGCAGAGCATC |  |
| qRT-PCR-*SmHPPR*-F | TGACTCCAGAAACAACCCACATT |  |
| qRT-PCR-*SmHPPR*-R | CCCAGACGACCCTCCACAAG |  |
| qRT-PCR-*SmRAS1*-F | CGAGATCGCCTACTCCAAGTTCAAG |  |
| qRT-PCR-*SmRAS1*-R | AGATGGCGTTACCGAAGTACCCC |  |
| qRT-PCR-*SmCYP98A14*-F | GGTCTGTACCGTCGTCCTCTTCTCC |  |
| qRT-PCR-*SmCYP98A14*-R | CCTTTTCCCAAATACCAGCCTTGT |  |
| qRT-PCR-*SmbZIP51*-F | TCGGAGACCTTCTTCCGCTT |  |
| qRT-PCR-*SmbZIP51*-R | TCGAAGAAATCCGCGTCCAC |  |
| qRT-PCR-*SmMYB53*-F | CGGAGGTGGAGAGAGCAAGA |  |
| qRT-PCR-*SmMYB53*-R | GAGCTGATTGCACCAACGGA |  |
| qRT-PCR-*SmActin*-F | AGCACCGAGCAGCATGAAGATT |  |
| qRT-PCR-*SmActin*-R | AGCAAAGCAGCGAACGAAGAGT |  |
