# Supplementary figures and images for: Molecular mechanism of SmMYB53 activates the expression of SmCYP71D375, thereby modulating tanshinone accumulation in Salvia miltiorrhiza
Source: Hortic Res. 2025 Feb 27;12(6):uhaf058. doi: 10.1093/hr/uhaf058 (PMC12017799; doi:10.1093/hr/uhaf058)

## MVA pathway

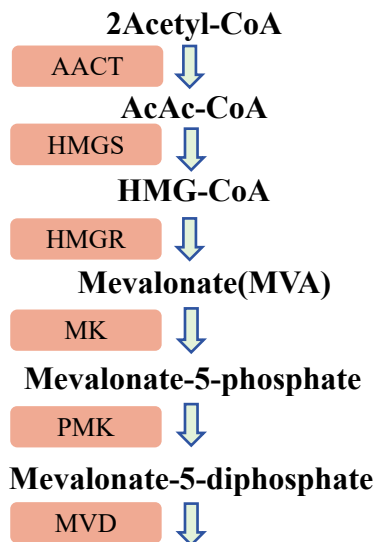

## MEP pathway

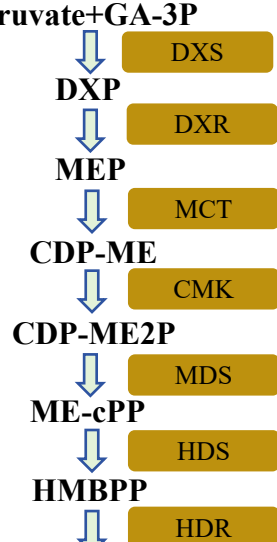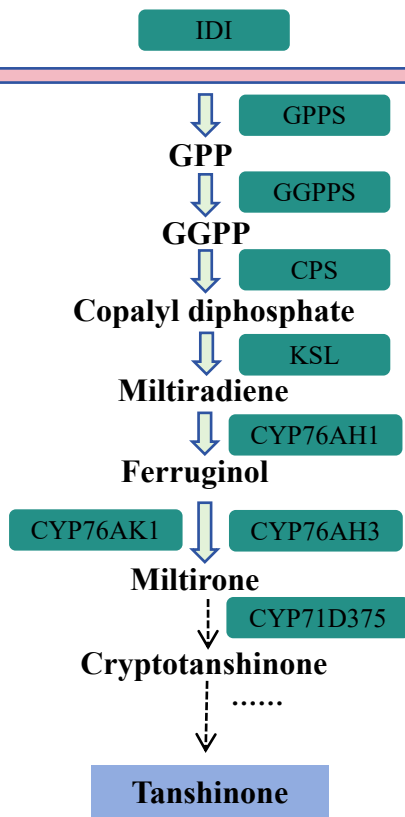

Supplement: Web_Material_uhaf058 [file web_material_uhaf058.zip › Supplementary Figure S1.pdf]

A

*SmMYB53*-OE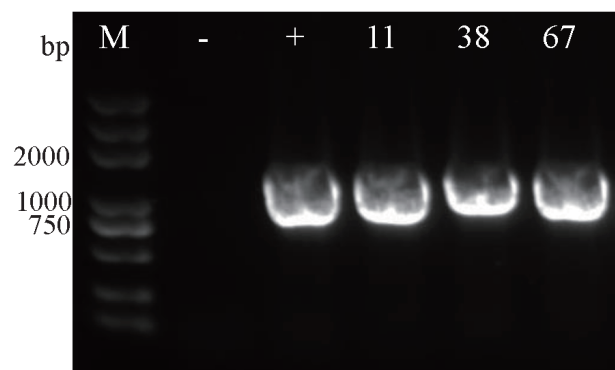

rolB

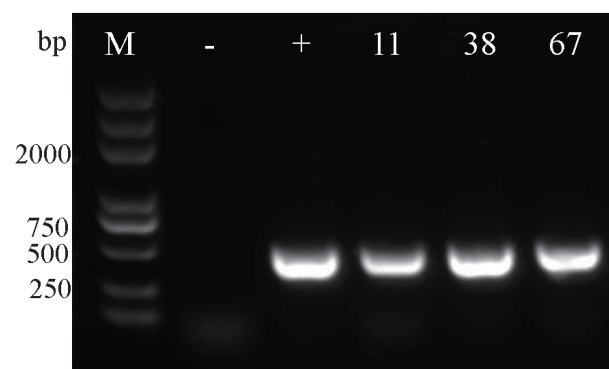

B

*SmMYB53*-RNAi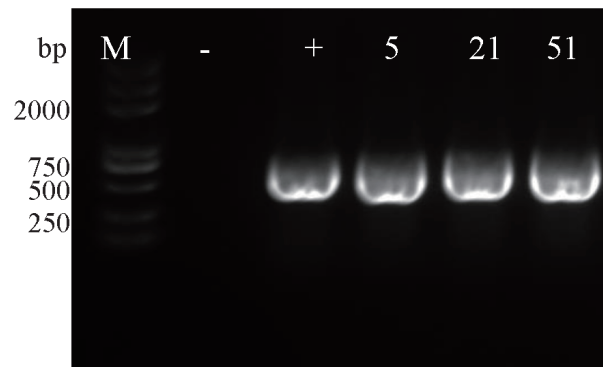

rolB

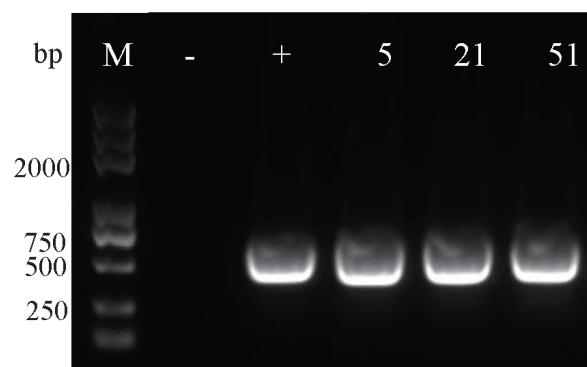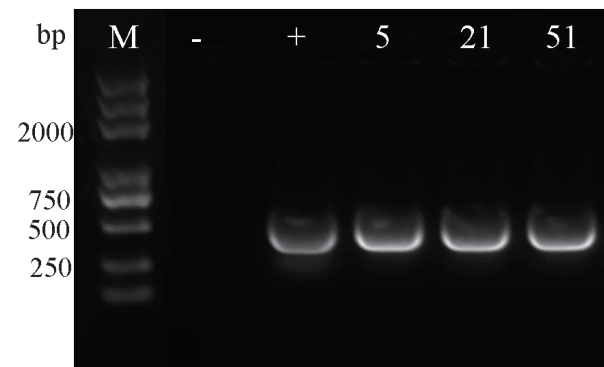

C

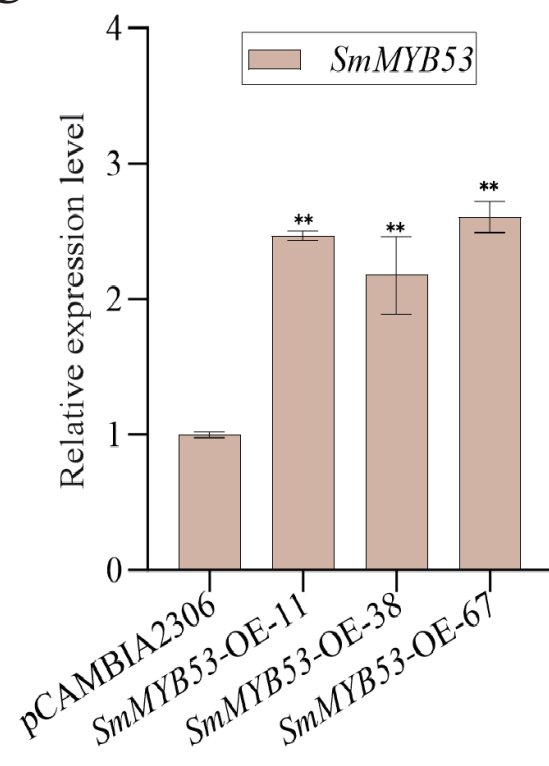

D

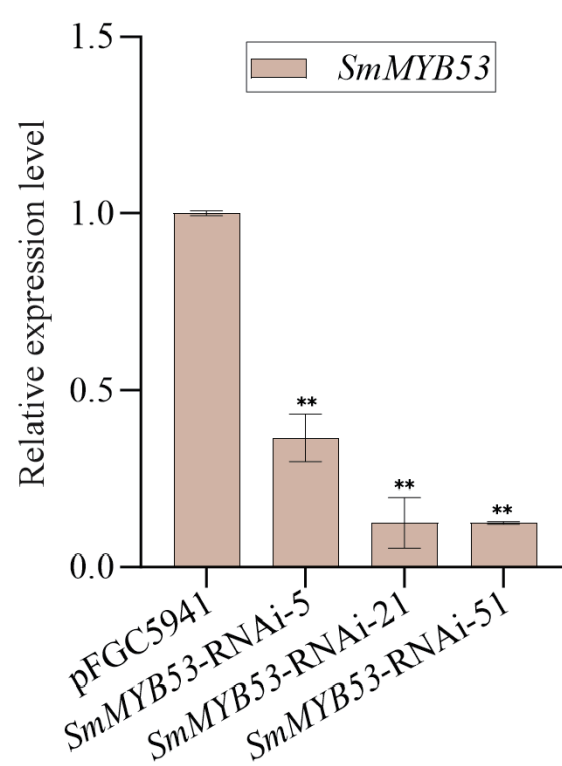

Supplement: Web_Material_uhaf058 [file web_material_uhaf058.zip › Supplementary Figure S3.pdf]

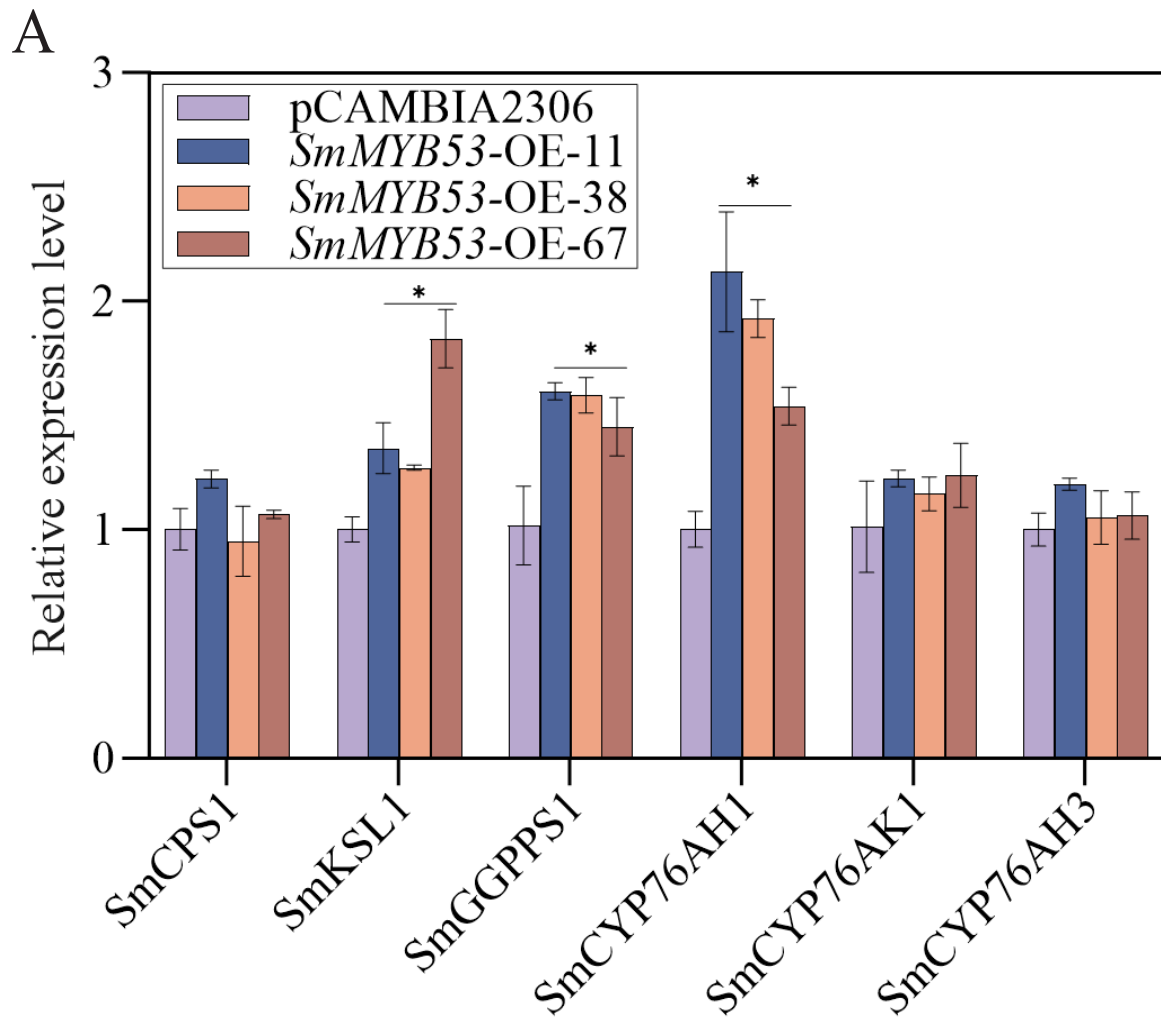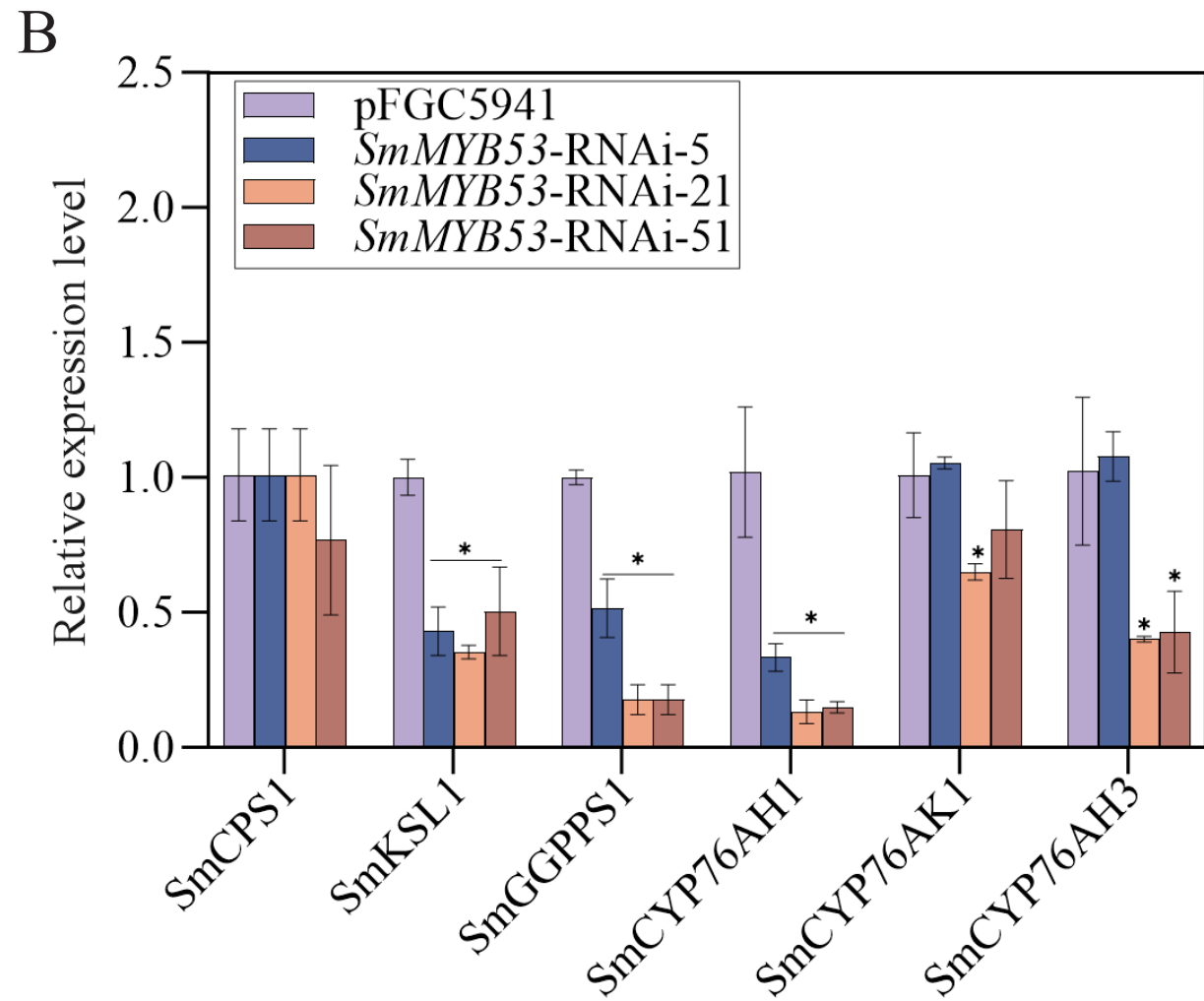

Supplement: Web_Material_uhaf058 [file web_material_uhaf058.zip › Supplementary Figure S4.pdf]

A

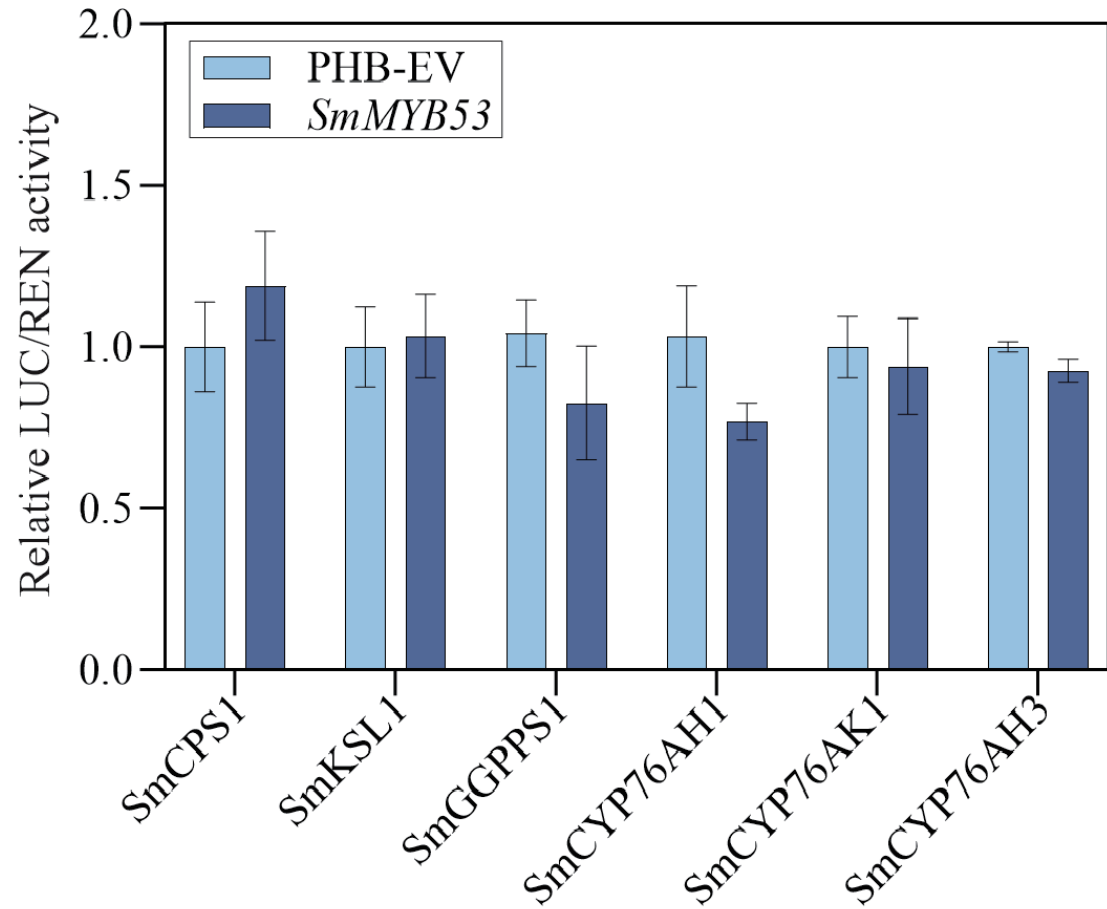

B

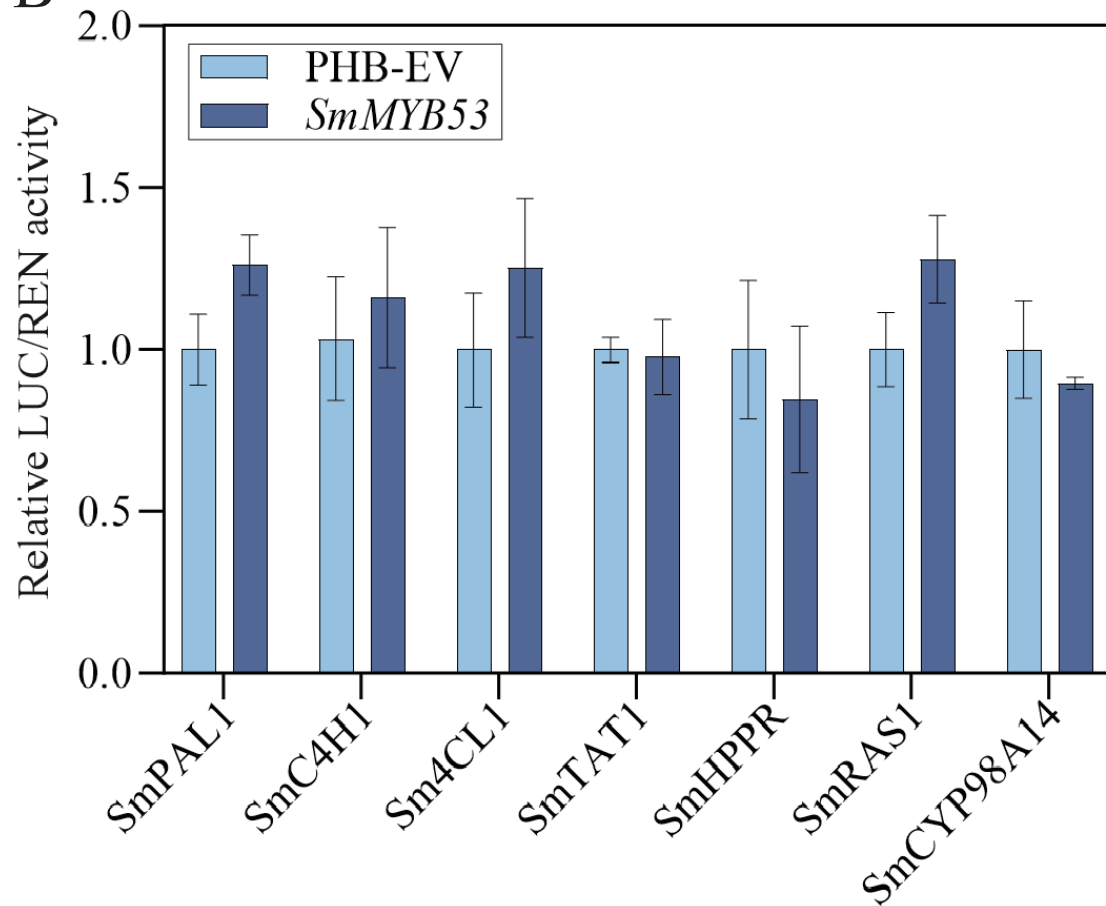

Supplement: Web_Material_uhaf058 [file web_material_uhaf058.zip › Supplementary Figure S5.pdf]

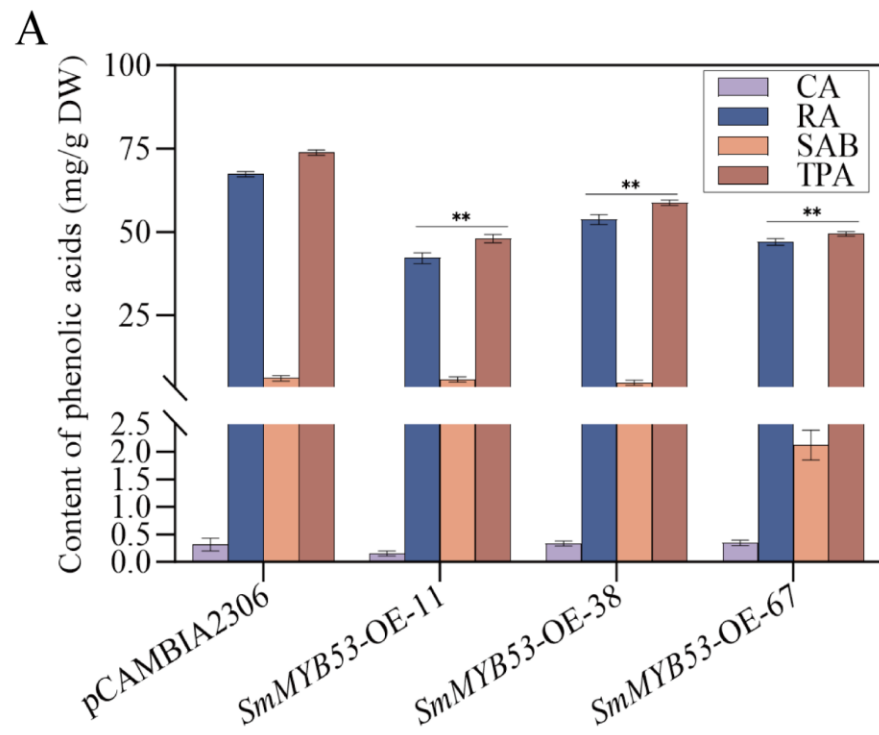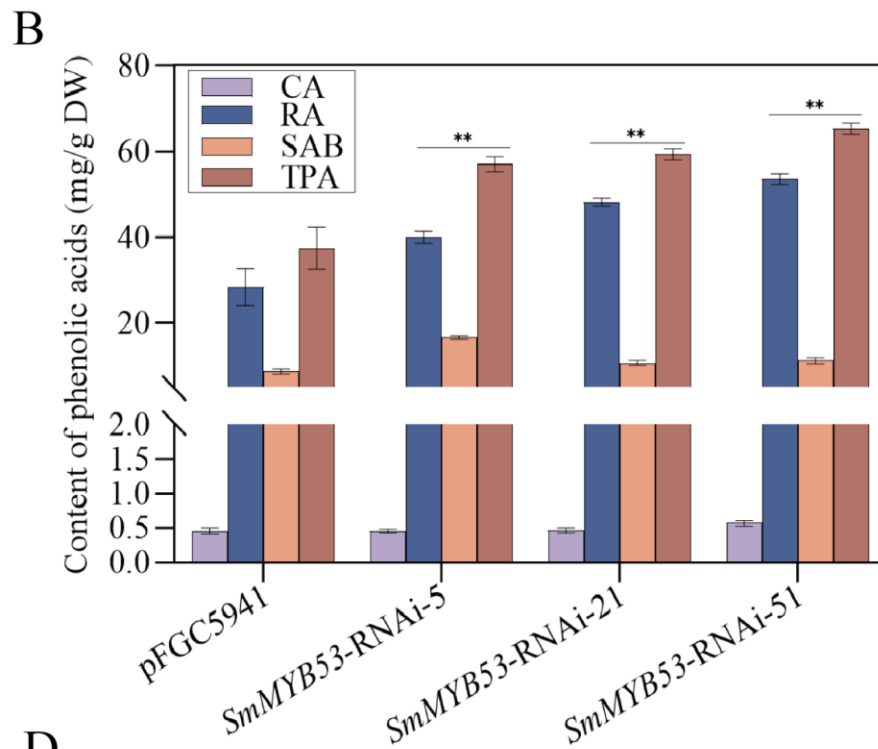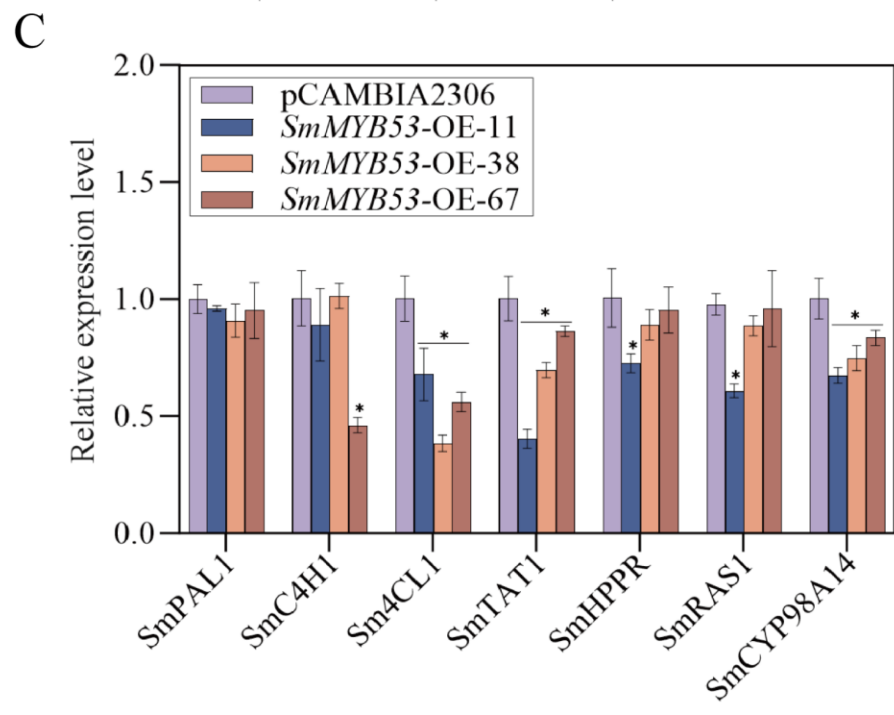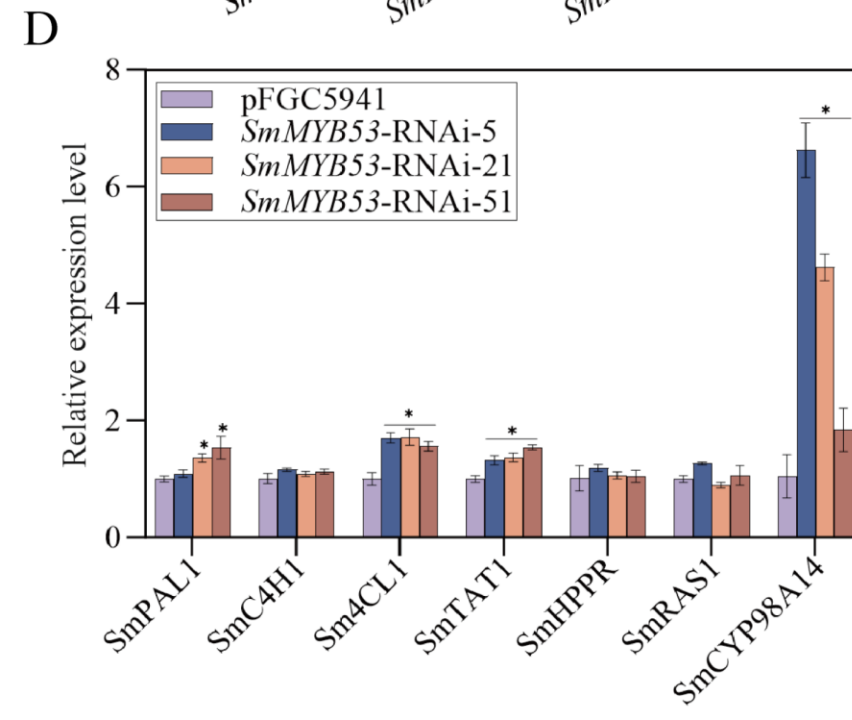

Supplement: Web_Material_uhaf058 [file web_material_uhaf058.zip › Supplementary Figure S6.pdf]

# A *SmMYB53*-OE

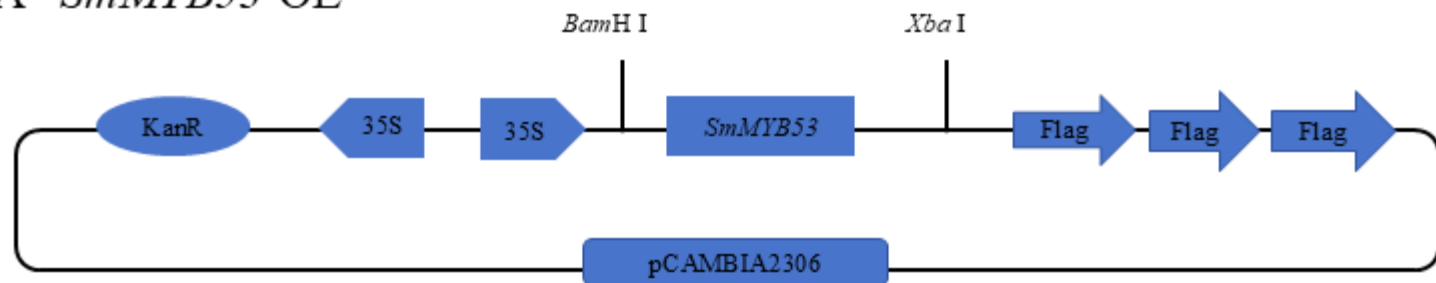

# B *SmMYB53*-RNAi

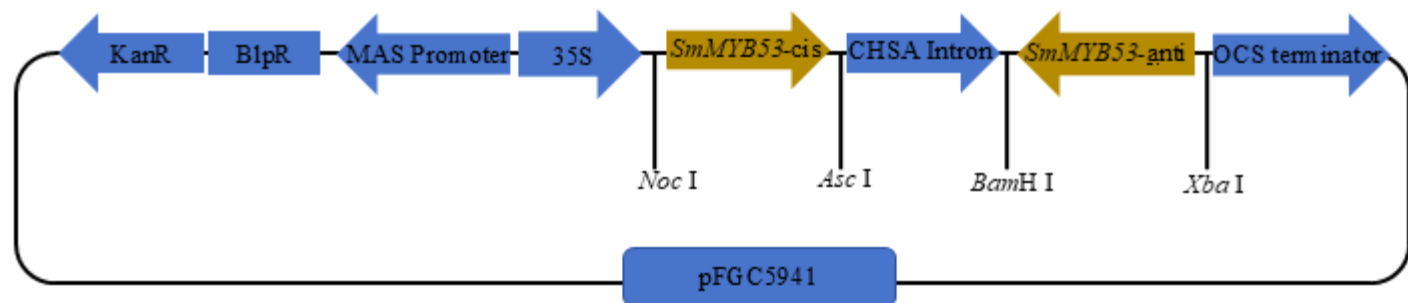

Supplement: Web_Material_uhaf058 [file web_material_uhaf058.zip › Supplementary Figure S7..pdf]
